# Host responses to *S. pneumoniae* in wild type and Mertk mutant mice

**DOI:** 10.1371/journal.pone.0320660

**Published:** 2025-04-16

**Authors:** Matthew K. McPeek, Jessica R. Martin, John C. Gomez, Yitong Li, Hong Dang, H. Shelton Earp, Claire M. Doerschuk

**Affiliations:** 1 Marsico Lung Institute, University of North Carolina at Chapel Hill, Chapel Hill, North Carolina, United States of America; 2 Department of Cell Biology and Physiology, University of North Carolina at Chapel Hill, Chapel Hill, North Carolina, United States of America; 3 Lineberger Comprehensive Cancer Center, Department of Medicine, University of North Carolina at Chapel Hill, Chapel Hill, North Carolina, United States of America; 4 Division of Pulmonary Diseases and Critical Care Medicine, Department of Medicine, University of North Carolina at Chapel Hill, Chapel Hill, North Carolina, United States of America; University of Colorado - Anschutz Medical Campus, UNITED STATES OF AMERICA

## Abstract

*Streptococcus pneumoniae* is the leading cause of community-acquired pneumonia. Mertk is a receptor tyrosine kinase and a member of the TAM family. It serves as an efferocytosis receptor involved in the recognition and removal of apoptotic debris by phagocytic cells, dampening the inflammatory response. Here we show that at 24h post-inoculation with *S. pneumoniae*, *Mertk*^*-/-*^ mice generated through homologous recombination and backcrossed (HRB-*Mertk*^*-/-*^ mice) have fewer bacteria present in their pneumonic lung than wild type mice. This enhanced clearance was not observed in *Mertk*^*-/-*^ mice generated by CRISPR technology. The enhanced clearance of HRB-*Mertk*^*-/-*^ mice was associated with fewer neutrophils and more IFNγ in the bronchoalveolar lavage, but was not prevented by a neutralizing IFNγ antibody. Mertk is highly expressed on alveolar macrophages. Transcriptomic changes observed in HRB-*Mertk*^*-/-*^ alveolar macrophages were associated with leukocyte activation, cellular motility, and response to stimulus, suggesting that they are primed for an inflammatory response. HRB-*Mertk*^*-/-*^ mice similarly had enhanced host defense pathways in *S. pneumoniae-*stimulated alveolar macrophages *in vitro* and in pneumonic lung tissue. However, HRB-*Mertk*^*-/-*^ alveolar macrophages demonstrated no defect in phagocytosis and acidification *in vivo*, and genes and gene sets describing phagocytic pathways were not enriched, suggesting that the enhanced clearance may be through alterations in the lung microenvironment. HRB-*Mertk*^*-/-*^ mice are reported to have a long 129P2 DNA insert (~645 genes) in chromosome 2 adjacent to Mertk, as well as other alterations at multiple sites. Thus, while Mertk deficiency may contribute to the enhanced bacterial clearance, it is not solely responsible, because the phenotype is not seen in the CRISPR-*Mertk*^*-/-*^ mice. The 129P2 DNA insert in the HRB-*Mertk*^*-/-*^ mice must be mediating at least some of this phenotype. Understanding the mechanistic differences and the means by which this 129P2 DNA insert enhances bacterial clearance remains critically important.

## Introduction

Bacterial pneumonias are a common disease and public health problem, by some criteria the most common cause of death, and result in high morbidity, mortality and cost to the health care system [1–7]. They are a frequent cause of ALI/ARDS and sepsis. Understanding resolution of infection requires integrated knowledge of many processes, including the clearance of neutrophils and recruited macrophages, development of the innate and adaptive immune response, and the repair of bronchiolar and alveolar epithelium. These processes can return the lungs to their normal structure and continue to serve effectively as a gas-exchanging organ.

Pneumonias induced by Gram-negative or -positive extracellular bacteria share aspects of pathology despite differences in mechanisms and particular features. The first line of defense is mucociliary clearance and resident alveolar macrophages, which are very effective against organisms in the airways and/or the alveoli and bronchioles, respectively. The innate immune system utilizes a system of pattern recognition receptors (PRRs) that detect pathogen-associated molecular patterns (PAMPs), which are distinct structures on bacteria, viruses and other pathogens [8,9]. If mucociliary clearance and resident macrophages are not sufficient, neutrophils are recruited, and edema develops due mostly to increased vascular permeability. Neutrophils recognize the presence of the pathogen either through the PRRs they express or through chemokines (IL-8/KC/MIP-2) and cytokines (IL-1, TNF) generated by alveolar macrophages or pulmonary epithelial cells. They sequester in the pulmonary capillaries at the infected site, beginning to migrate into lung tissue within 4 h after bacterial challenge. Soon after neutrophils begin to arrive, macrophage numbers increase, followed by other immune cells, including CD4 and CD8 T cells, NK cells, NKT cells, dendritic cells, innate lymphoid cells, B cells and regulatory T cells, which are recruited to the alveoli and perivascular tissue. Neutrophils die through apoptosis, necrosis, necroptosis and NETosis and are cleared, often through efferocytosis. Alveolar epithelial type 2 (AT2) cells proliferate, pass through transitional phases, and differentiate to become Type 1 (AT1) cells, repairing the epithelial surface of the airspaces.

*S. pneumoniae* is a Gram-positive α-hemolytic diplococcus, usually surrounded by a thick polysaccharide capsule, which expresses pneumolysin and several adhesins. It remains the most common cause of community-acquired pneumonias[1,4,7]. It is also an important cause of nosocomial pneumonias and secondary bacterial infections following viral infections. Although all the host PRRs that any organism recognizes are not known, *S. pneumoniae* is well documented to recognize extracellular TLR2 and 4, intracellular TLR9, STING and NOD2 [10,11]. It also interacts with the complement system in novel ways [12].

Mertk (myeloid-epithelial-reproductive proto-oncogene tyrosine kinase) is a receptor tyrosine kinase and a member of the TAM family, which also includes Tyro3 and Axl [13,14]. Mertk consists of an extracellular ligand binding domain, a transmembrane region, and an intracellular protein-tyrosine kinase domain containing the sequence KWIAIES [15]. Mertk is activated by the ligands, growth arrest-specific gene 6 (Gas6) and Protein S (Pros1), which bind the extracellular domain and induce auto-phosphorylation and downstream signal transduction pathways[16,17]. While ligand binding alone can induce auto-phosphorylation, the most robust signaling occurs when Gas6 or Pros1 function as a bridge molecule to phosphatidylserine expressed on the surface of a second, apoptotic cell. Phosphatidylserine, which is restricted to the inner leaflet of the plasma membrane of healthy cells, is presented on the outer leaflet during apoptosis and serves as an “eat-me” signal recognized by phagocytes [18]. Thus, apoptotic cells, which express phosphatidylserine on their membrane, are particularly effective at stimulating Mertk, inducing efferocytosis. Deficiency of Mertk or other TAM receptors in murine models leads to the progressive accumulation of apoptotic debris in homeostatic tissue and impaired clearance of apoptotic cells, such as neutrophils, during the resolution phase of the inflammatory response [14,15,19]. However, Mertk deficiency by itself does not diminish phagocytosis of *Listeria monocytogenes* or microbeads [19].

This study tested the hypothesis that Mertk regulates the inflammatory process induced by *S. pneumoniae*. These data show that Mertk is highly expressed on alveolar macrophages in the naïve lung. *Mertk*^*-/-*^ mice generated through homologous recombination and backcrossed (HRB-*Mertk*^*-/-*^ mice). When presented with a bacterial challenge, the HRB-*Mertk*^*-/-*^ mice had fewer bacteria present in their pneumonic lung than wild type mice. This defect was not observed when mice lacking Mertk generated directly in C57Bl/6 by CRISPR technology (CRISPR-*Mertk*^*-/-*^ mice) were studied. The enhanced clearance observed in HRB-*Mertk*^*-/-*^ mice was associated with fewer neutrophils and more IFNγ in the bronchoalveolar lavage; however, a neutralizing IFNγ antibody did not prevent the enhancement of clearance. HRB-*Mertk*^*-/-*^ mice had alterations in the transcriptome of naïve alveolar macrophages that promoted their response to external stimuli when these cells were exposed to *S. pneumoniae in vitro.* The lack of a defect in CRISPR-*Mertk*^*-/-*^ mice suggests that the recognized 129P2 DNA insert and the residual regions of the 129 strain in HRB-*Mertk*^*-/-*^ mice account for the enhanced clearance [20,21]. Because means of enhancing bacterial clearance may be critically important as a therapeutic approach in patients with pneumonia, we pursued these studies due to their high potential significance.

## Materials and methods

### Mice

All animal studies were performed in accordance with the Public Health Service Policy on Humane Care and Use of Laboratory Animals*.* All procedures received prior approval by the University of North Carolina Institutional Animal Care and Use Committee. Animals were housed in ventilated cages, and colonies were maintained within a pathogen-free facility at the University of North Carolina (UNC). C57Bl/6J wild type mice were originally purchased from The Jackson Laboratory (stock no. 000664, Bar Harbor, Maine). Male or female mice, 7-9 weeks of age were utilized for all studies. At the time point indicated for each study, animals were euthanized by lethal overdose of inhaled isoflurane. All efforts were made to minimize distress or suffering.

Mertk deficient mice were generated by homologous recombination that introduced a neomycin resistance cassette into exon 18, the last exon encoding the 3’ end of the kinase domain [22], and backcrossed to a C57Bl/6J background [19]. These animals are designated as homologous recombinants and backcrossed (HRB)-*Mertk*^*-/-*^ mice.

A new knockout of Mertk was generated using CRISPR/Cas9 technology (UNC Mouse Models Core, Dale Cowley, PhD, Director) for one of our laboratories (HSE). A Mertk flox allele was inserted using CRISPR/Cas9 directly into the genome of C57Bl/6J mice. Following creation of the fl/fl mouse, embryos with the floxed allele were exposed to cell-permeable Cre recombinase protein to delete this allele, generating a whole-body constitutive knockout Mertk allele. These *Mertk* null mice are designated CRISPR-*Mertk*^*-/-*^ mice.

### Bacterial pneumonia

*Streptococcus pneumoniae* (*S. pneumoniae;* serotype 19, ATCC 49619) was purchased from American Type Culture Collection. Bacteria were grown overnight on BD trypticase soy agar containing 5% defibrinated sheep blood (VWR International, Cat. 90001-282) at 37°C with 5% CO_2_. Bacteria were suspended in phosphate-buffered saline (PBS), and the concentration was estimated based on spectrophotometric interference at 600nm (~0.175 AU). Animals were anesthetized with tribromoethanol (0.25mg/g body weight) prior to intratracheal instillation of bacterial suspension into the left lung by 24-gauge catheter (2.27 µ L/g body weight). Colony forming units (CFUs) of the bacterial suspensions were subsequently determined by serial dilution on agar plates.

### Bacterial clearance

24 hours post-inoculation, mice were euthanized using lethal overdose of inhaled isoflurane. The left lung was harvested using sterile technique and homogenized in 1 mL ice-cold PBS using a hand-held homogenizer. CFUs were determined by plating serial dilutions of the lung homogenate as described above. In separate studies, the location of the bacteria was determined. Bronchoalveolar lavage was performed as described below, and the numbers of *S. pneumoniae* present in the lavage fluid, the cell-free lavage fluid following centrifugation, the cell pellets, and the lavaged lung tissue were quantified by counting CFUs in serial dilutions.

### Quantification of leukocyte phagocytosis in vivo

pHrodo *S. aureus* BioParticles (Cat.P35367, ThermoFisher) were reconstituted in PBS (2.27mg/mL) and evenly dispersed, following the manufacturer’s recommendation. Animals were anesthetized using inhaled isoflurane, and 100 µ L of bioparticles were administered via oropharyngeal aspiration. One hour after instillation, animals were euthanized by lethal overdose of inhaled isoflurane. Both lungs were lavaged, instilling 0.7 mL PBS containing 2mM EDTA, aspirating the fluid, and repeating the process 10 times, totaling 7 mL. The cell pellet was collected by centrifugation at 400 g for 10 min. Total cell counts were determined by hemocytometry. Alveolar macrophages were identified by immunostaining using antibodies against cell-specific surface markers and flow cytometry, as described below. Intracellular *S. aureus* bioparticles were identified by fluorescence.

### Preparation of single-cell lung digest for flow cytometry

Animals were euthanized by lethal overdose of inhaled isoflurane. The right bronchus was tied off with suture, and the left lung was instilled with 0.5 mL of RPMI media containing 5mg/mL collagenase I (Worthington Biochemical Corporation, Cat. LS004197) and 1mg/mL deoxyribonuclease I (Worthington, Cat. LS002140) followed by 0.3mL 1% (wt/vol) low melting agarose (Invitrogen) as a tracheal plug. Lungs were incubated at 37°C for 30 minutes, minced, and passed through an 18-gauge needle. Cell suspensions were filtered through a 100 µ M filter prior to red blood cell lysis using ACK lysis buffer (ThermoFisher, Gibco, Cat. A1049201).

### Bronchoalveolar lavage

Bronchoalveolar lavage (BAL) was performed on naïve controls, PBS-instilled controls, or pneumonic mice by tying off the right bronchus, instilling 0.4 mL ice-cold PBS containing 2mM ethylenediaminetetraacetic acid (EDTA), aspirating the fluid, and repeating the process 5 times, totaling 2 mL. The cell pellet was collected by centrifugation at 400 *g* x 10 min. Total cell counts were determined by hemocytometer. Differential cell counts were obtained from cytospins stained with Hema 3 Stat Pack (Fisher Scientific, Cat. 22-122911). Cell pellets and aliquots of cell-free BAL fluid were flash frozen and stored at -80°C.

### Flow cytometry analysis

Single cell lung digests (2.5 x 10^6^ cells) or BAL cells (3.5 x 10^5^ cells) were suspended in buffer (PBS containing 1.6% BSA and 2mM EDTA) containing rat anti-mouse FcγRIII/II receptor block (BD Biosciences, Cat. 553142) for 20 minutes. After Fc blockade, antibodies against cell surface markers were added for 30 minutes. See S1 Table in S1 File for antibody source, clone, and concentrations. Flow cytometry was performed using a Cytoflex flow cytometer and analyzed using CytExpert (Beckman Coulter). Neutrophils, macrophage subpopulations, and natural killer cells were identified through sequential gating as described in S1 Fig in S1 File.

Immune cells were identified as:

1. Neutrophils: CD45^ + ^Ly6G^+^CD64^-^
2. Alveolar macrophages: CD45^ +^ Ly6G^-^ CD64^ +^ SiglecF^ +^ Ly6C^-^
3. Interstitial macrophages: CD45^ +^ Ly6G^-^ CD64^ +^ SiglecF^-^ Ly6C^-^
4. Inflammatory macrophages: CD45^ +^ Ly6G^-^ CD64^ +^ SiglecF^-^ Ly6C^ + ^
5. Natural killer cells: CD45^ +^ Nk1.1^ + ^

### Quantitation of neutrophil extracellular traps (NETs)

NETs were quantified in the BAL fluid of naïve or pneumonic female mice 24 hours post inoculation using a modified sandwich ELISA identifying the amount of DNA that bound myeloperoxidase. Following collection and prior to centrifugation, aliquots of BAL fluid were flash frozen in liquid nitrogen. Myeloperoxidase in the BAL fluid (100 µ L) was bound using the Myeloperoxidase Mouse ELISA Kit (ThermoFisher, Cat. EMMOPO) according to the manufacturer’s recommendations. Bound DNA was subsequently detected using the anti-DNA-POD-HRP antibody from the Cell Death Detection ELISA (Roche, Cat. 11544675001). Data are reported as normalized absorbance values when compared to negative control wells.

### Analysis of mediators in BAL fluid

Aliquots of BAL fluid were analyzed using the Bio-Plex Pro Mouse Cytokine Grp I Panel 23-plex (Bio-Rad Laboratories, Cat. M60009RDPD), the Interferon gamma (IFNG) Mouse ELISA Kit (Abcam Cat. 100689), or the Pierce BCA Protein Assay Kit (ThermoFisher, Cat. 23225).

### Interferon-gamma (IFNγ) neutralization

Blockade of IFNγ was accomplished by treating female wild type and *HRB-Mertk*^*-/-*^ mice with a blocking anti-mouse IFNγ antibody intraperitoneally an hour before inoculation and intratracheally with the inoculum [23,24]. Purified, ultra-low endotoxin/azide-free anti-mouse IFNγ antibody (cat #505847) and isotype-matched control (cat #400457) were purchased from BioLegend. One hour prior to inoculation, female mice received an intraperitoneal injection of 200µg anti-IFNγ antibody in 200 µ L. Two control groups were included, one given 200µg isotype control antibody and the other given PBS. At the time of inoculation, the inoculum was prepared by combining a concentrated *S. pneumoniae* suspension (~0.5 AU as described above) with either anti-IFNγ antibody, isotype control or PBS, so that the final concentration of antibody in the inoculum was 100µg/50 µ L. Pneumonia was induced as described above.

### Ex vivo alveolar macrophage culture and exposure to *S. pneumoniae*

Alveolar macrophages were isolated from naïve male mice by bilateral, whole lung lavage. Animals were euthanized by lethal overdose of inhaled isoflurane immediately prior to cannulating the trachea and inflating the lung with 1mL room-temperature PBS containing 2mM EDTA. The lavage was withdrawn, and the process was repeated 10 times, totaling 10mL. BAL samples were maintained at room temperature throughout the processing procedure. Cells were pelleted, 400*g* x 10min, and washed two times in 5mL PBS containing no EDTA. Cells were resuspended in RPMI medium containing 10% FBS (medium) at 37°C. Cells (2.2x10^5^) from either genotype were plated in duplicate using Nunc Lab-Tek II Chamber Slides (ThermoFisher, cat# 154534) and incubated at 37°C with 5% CO_2_. Non-adherent cells were removed by gentle washing 3 hours after plating, and cultures were incubated overnight (24 hours total). *S. pneumoniae* was collected from agar plates as described above and suspended in RPMI medium (OD: 600nm ~ 0.175 AU). Cells were stimulated with 200 µ L medium containing *S. pneumoniae* or medium only (unstimulated) and incubated for 4 hours. The multiplicity of infection was determined to be 30.5 ±  1.7 (mean ±  SEM) viable *S. pneumoniae* per macrophage. Following stimulation, the medium was removed, and total RNA was immediately collected using the Quick-RNA Microprep Kit (Zymo Research, Cat. R1050). The RNA integrity numbers (RINs) isolated from unstimulated cells were 8.2-10.0, and the RINs from *S. pneumoniae-*stimulated cells were 4.9-7.2, as determined by Agilent bioanalyzer.

### RNA isolation from naïve alveolar macrophages and naïve and pneumonic left lung tissue

Alveolar macrophages utilized for RNA sequencing studies were isolated from naïve animals by bilateral, whole lung lavage. Mice were euthanized by lethal overdose of inhaled isoflurane immediately prior to cannulating the trachea and inflating the lungs with 1 mL ice-cold PBS containing 2mM EDTA. The lavage fluid was withdrawn, and the process was repeated 7 times, totaling 7 mL. Cell counts and differential cell analysis were performed as described above. Cell pellets were flash frozen and stored at -80°C. Left lung tissues were collected from additional male mice with 24h *S. pneumoniae* pneumonia (*n = * 8, two independent experiments) following overdose of inhaled isoflurane. Controls were the left lung of naïve mice (n =  5). Tissue was immediately flash frozen and stored at -80°C. Total RNA was isolated from BAL cell pellets or left lung tissue using the Quick-RNA Microprep kit (Zymo Research) or the miRNeasy Mini Kit (Qiagen), respectively. All samples had an RNA integrity number >  8.0 when assessed using the Agilent RNA 6000 Nano Kit (Agilent Technologies). Total RNA was submitted to the indicated commercial vendor for library preparation and RNA sequencing using the Illumina platform. RNA sequencing data of naïve alveolar macrophages represent analysis of two independent sequencing runs (Novogene). Data describing naïve and pneumonic lung tissue represent the analysis of a single sequencing (Azenta).

### Transcriptomic analyses of alveolar macrophages and lung tissue

Raw sequence reads in fastq format were mapped to the current reference genome (naïve and cultured alveolar macrophage reference genome: GRCm38, GENCODE vM25; lung tissue reference genome: GRCm39, GENCODE vM29) using the aligner STAR [25]. Primary gene annotation was determined from GENCODE. Gene expression quantification was performed using StringTie transcriptome assembler [26]. Beginning with the raw count data, genes were filtered based on minimal expression, > 50 total counts across all samples. Data were normalized across all samples using voom [27], and differential gene expression was determined using the linear models using the Bioconductor R package, *limma* [28]. Gene sets and pathways enrichment analysis (GSEA, [29]) was performed using the Bioconductor R package *fgsea* [30] based on all expressed genes ranked by log2 fold change, determined by differential expression analyses. Collections of gene sets were downloaded from current Gene Ontology (GO, [31]) biological processes and Reactome [32]. A differentially expressed gene (DEG) was defined using the following criteria: 1) log2 average expression among all samples greater than zero, 2) false discovery rate adjusted p value less than 0.05 (FDR < 0.05), 3) fold change greater than ±  1.5. Venn diagrams were generated using InteractiVenn [33]. Clustering heatmaps were generated for DEGs from transcripts per million (TPM) values using the Bioconductor R package *ComplexHeatmap* [34]. Patterns of gene expression were determined by hierarchical clustering of DEGs followed by cutree analysis. Over-representation analysis was performed using DEGs within each cluster to identify GO terms describing associated gene sets and pathways.

### Accession number

The RNA sequencing data were deposited into the Gene Expression Omnibus (http://www.ncbi.nlm.nih.gov/geo/) under the series accession number GSE210551.

### Statistical analysis

Except for comparison of gene expression, all statistical analyses were performed using GraphPad Prism 8 software (GraphPad Software Inc.). The statistical tests used for each comparison are detailed in the figure legends. *p < * 0.05 was considered statistically significant.

### Genes predicted to be within the 129P2 DNA insert of HRB-*Mertk*^-/-^ mice

Complimentary DNA sequences for the primers used to define the 129P2 insertion on chromosome 2 by Akalu *et al.* ([21]; D2Mit206-R: 5’-GCCCTAATCATTAGTGTCTGTTATCAT-3’ and D2Mit168-R: 5’-CCCAAAACAATAGCAGGAACA-3’) were identified. The sequences were mapped to the GRCm38/mm10 reference genome using the BLAT alignment tool (https://genome.ucsc.edu/index.html; [35]). The region was defined as chr2:106,909,939 - 144,822,198. A list of known genes was generated using the UCSC Table Browser [36] and cross referenced to DEGs identified in transcriptomic analysis of naïve alveolar macrophages and lung tissue.

## Results

### Mertk is expressed primarily on alveolar macrophages

**Fig 1:**
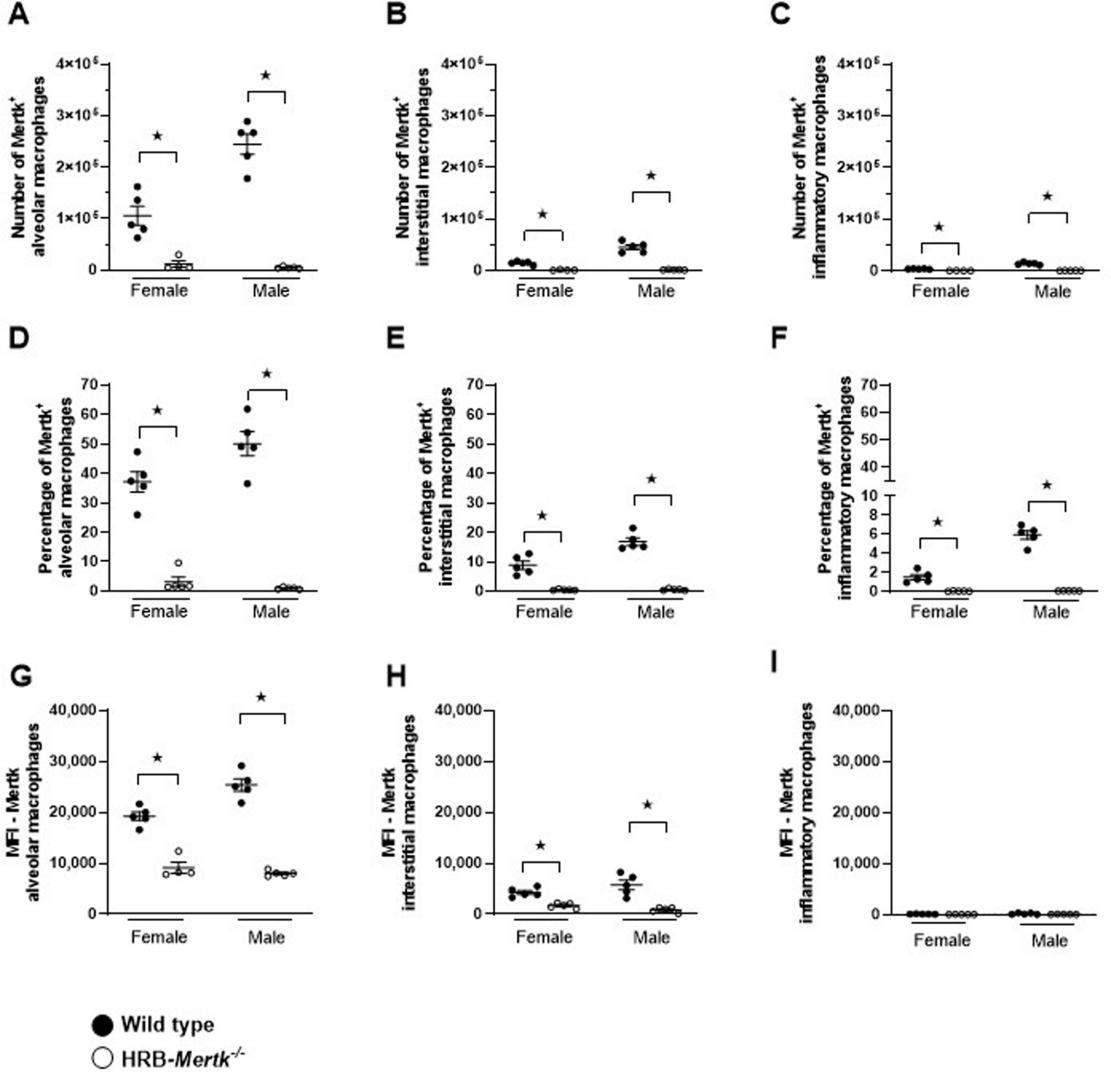
Mertk is expressed primarily on alveolar macrophages. The number of Mertk^ +^ macrophages in left lung homogenates of naïve HRB-*Mertk*^*-/-*^ and wild type mice were determined by flow cytometry. The number of Mertk^ +^ (**A**) alveolar, (**B**) interstitial, and (**C**) inflammatory macrophages was quantified. The (**D–E**) percentage of Mertk^ +^ cells and (**G–I**) median fluorescence intensity (MFI) of fluorescently-labeled anti-Mertk antibody staining was determined for each macrophage subpopulation. Data were collected from 2 independent experiments and are expressed as the mean ±  SEM*.* Significant differences determined by one-way ANOVA followed by Tukey’s multiple comparison test. ★ *p* <  0.05.

To compare the expression of Mertk on myeloid cells, we evaluated the number of Mertk^ +^ events within each macrophage subpopulation, neutrophils and natural killer cells. The threshold for Mertk^ +^ events was defined both by the use of fluorescence-minus-one control wells included in each experiment and HRB-*Mertk*^*-/-*^ mice, which yielded the same threshold. We identified Mertk^ +^ alveolar macrophages in wild type females and males (Fig 1A) and found these cells represented 34.1 ± 3.4% and 50.0 ± 4.1% of the subpopulation respectively (Fig 1D). There were fewer Mertk^ +^ interstitial macrophages than alveolar macrophages (Fig 1B), and a smaller percentage of interstitial macrophages expressed Mertk in both wild type females (8.9 ± 1.4%) and males (16.9 ± 1.3%; Fig 1E). Very few Mertk^ +^ inflammatory macrophages were identified in wild type females or males (Fig 1C), representing 1.5 + 0.3% and 5.9 ± 0.4% of the subpopulation respectively (Fig 1F).

The amount of Mertk on each macrophage subpopulation was estimated by the median fluorescence intensity (MFI) of fluorescently-labeled anti-Mertk antibody staining. Alveolar macrophages had the highest MFI in both female (19,245 ± 829) and male (25,369 ± 1187) wild type mice (Fig 1G). The MFI was lower on interstitial macrophages of wild type females (4,288 ± 389) and wild type males (5,749 ± 935; Fig 1H). The MFIs of wild type inflammatory macrophages were not statistically different from those of HRB-*Mertk*^*-/-*^ mice (Fig 1I). Mertk was also not detected on neutrophils or natural killer cells (S2 Fig in S1 File).

### HRB- but not CRISPR-*Mertk*^*-/-*^ mice have enhanced clearance of S. pneumoniae 24 hours post inoculation

To test our hypothesis that Mertk modulates host defense during *S. pneumoniae* pneumonia, we measured bacterial clearance in HRB-*Mertk*^*-/-*^ and wild type female and male mice. The number of inoculated bacteria was based on body weight. The original body weights and thus the inoculum CFUs were similar between genotypes (S2 Table in S1 File). Both genotypes and sexes lost about 10% of their original weight 24 hours post inoculation (S2 Table in S1 File).

**Fig 2:**
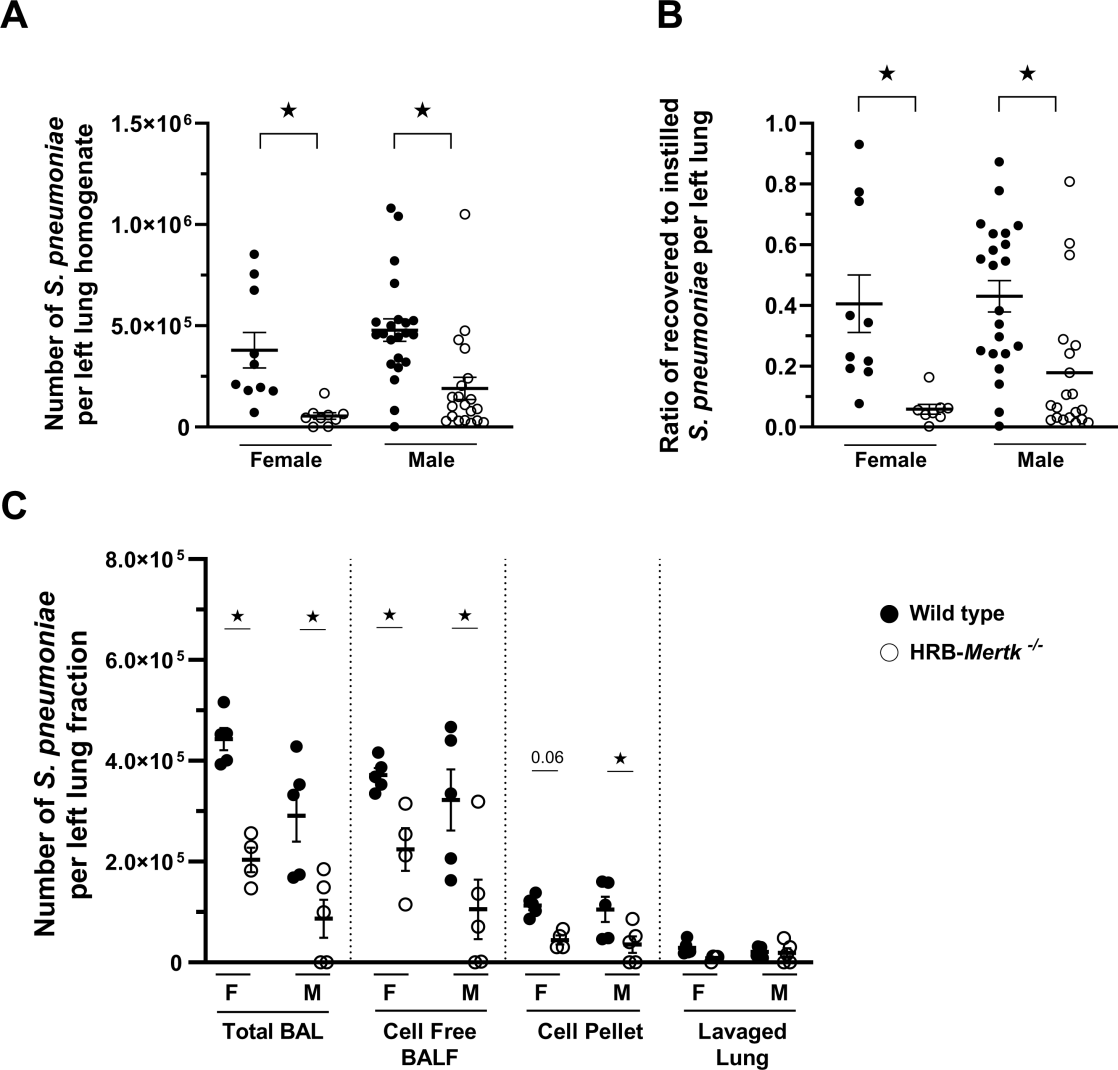
HRB-*Mertk*^*-/-*^ mice have greater clearance of *S. pneumoniae* 24 hours post inoculation. Bacterial clearance was assessed in HRB-*Mertk*^*-/-*^ and wild type male and female mice 24 hours post inoculation. The number of inoculated bacteria was based on body weight (2.20 ± 0.32x10^7^ CFU/mL). The (**A**) number of bacteria per left lung homogenate and the (**B**) ratio of recovered to instilled bacteria were determined, *n* =  9-22 per group. In a separate experiment, the (**C**) location of viable bacteria within the lung was determined by plating serial dilutions of specific lung fractions (*n* =  4-5 per group). Data represent mean ±  SEM. Significant differences determined by one-way ANOVA followed by Tukey’s multiple comparison, ★*p* <  0.05.

Bacterial clearance was quantified 24 hours after inoculation. Surprisingly, fewer viable bacteria were present in the lungs of both female and male HRB-*Mertk*^*-/-*^ compared to wild type mice (Fig 2A). The ratio of recovered to instilled *S. pneumoniae* was also significantly lower in HRB-*Mertk*^*-/-*^ mice compared to wild type mice of both sexes (Fig 2B). Thus, HRB-*Mertk*^*-/-*^ mice have enhanced clearance of *S. pneumoniae* by 24 hours.

To determine the location of viable bacteria in the lung 24 hours post-inoculation, the number of bacteria was quantified in the total BAL fluid, cell-free BAL fluid, cell pellet and lavaged lung homogenate after 24 hours. In both genotypes, approximately 70-80% of viable bacteria were detected in cell-free BAL fluid and 16-23% associated with the BAL cell pellet (Fig 2C). Similar to the clearance data obtained from left lung homogenates, HRB-*Mertk*^*-/-*^ mice had significantly fewer viable bacteria present in both the cell-free BAL fluid and the cell pellet when compared to wild type animals. Interestingly, relatively few bacteria (3-10%) were recovered in homogenized lung tissue following the BAL procedure, and no statistically significant differences were found between genotypes (Fig 2C). No differences were identified between sexes.

**Fig 3:**
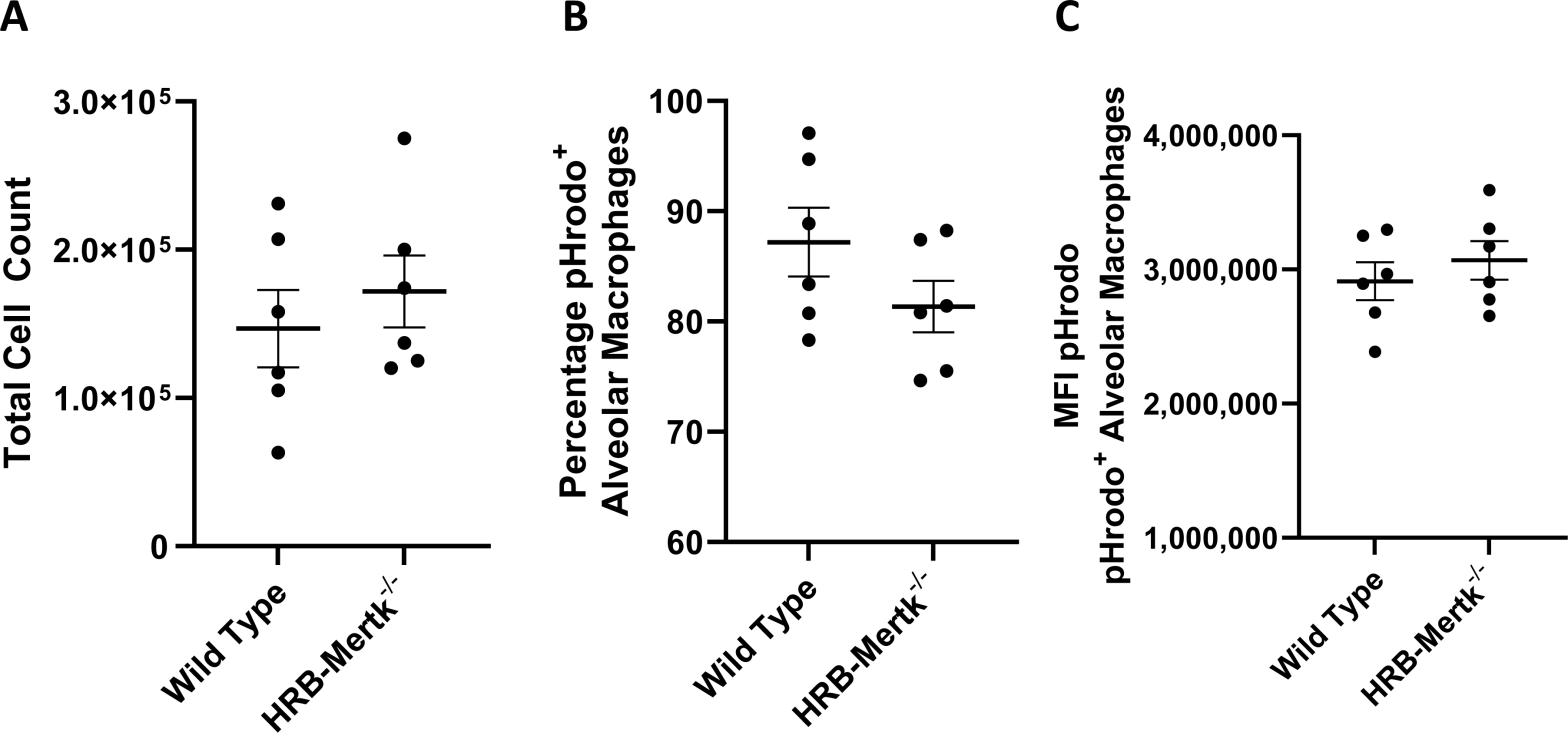
Alveolar macrophages from HRB-*Mertk*^*-/-*^ mice show no enhancement in phagocytosis *in vivo.* Clearance of pHrodo-labeled *S. aureus* bioparticles was assessed in HRB-*Mertk*^*-/-*^ and wild type male mice 1 hour post inoculation. **(A)** The number of alveolar macrophages did not differ between genotypes. The (**B**) percentage of alveolar macrophages and the (**C**) median fluorescent intensity (MFI) of pHrodo-labeled *S. aureus* bioparticles also did not differ between genotypes. Data represent mean ±  SEM, ****n**** =  6 per group. Groups were compared using a T-test.

To determine if phagocytosis by alveolar macrophages was the mechanism through which bacterial clearance was enhanced, pHrodo *S. aureus* bioparticles were instilled into the lungs of HRB-*Mertk*^*-/-*^ and wild type mice. These particles fluoresce only after phagocytosis and acidification by lysosomes, and thus are an excellent way to measure phagocytosis [37]. After 1 hour, the airspaces were lavaged, and flow cytometry was performed to identify alveolar macrophages and *S. aureus* bioparticles. There was no difference between genotypes in the number of alveolar macrophages within the BAL fluid, the percentage of these macrophages that phagocytosed and acidified *S. aureus* bioparticles, or the median fluorescent intensity (MFI) of pHrodo-labeled *S. aureus* bioparticles within the macrophages between genotypes (Fig 3).

**Fig 4:**
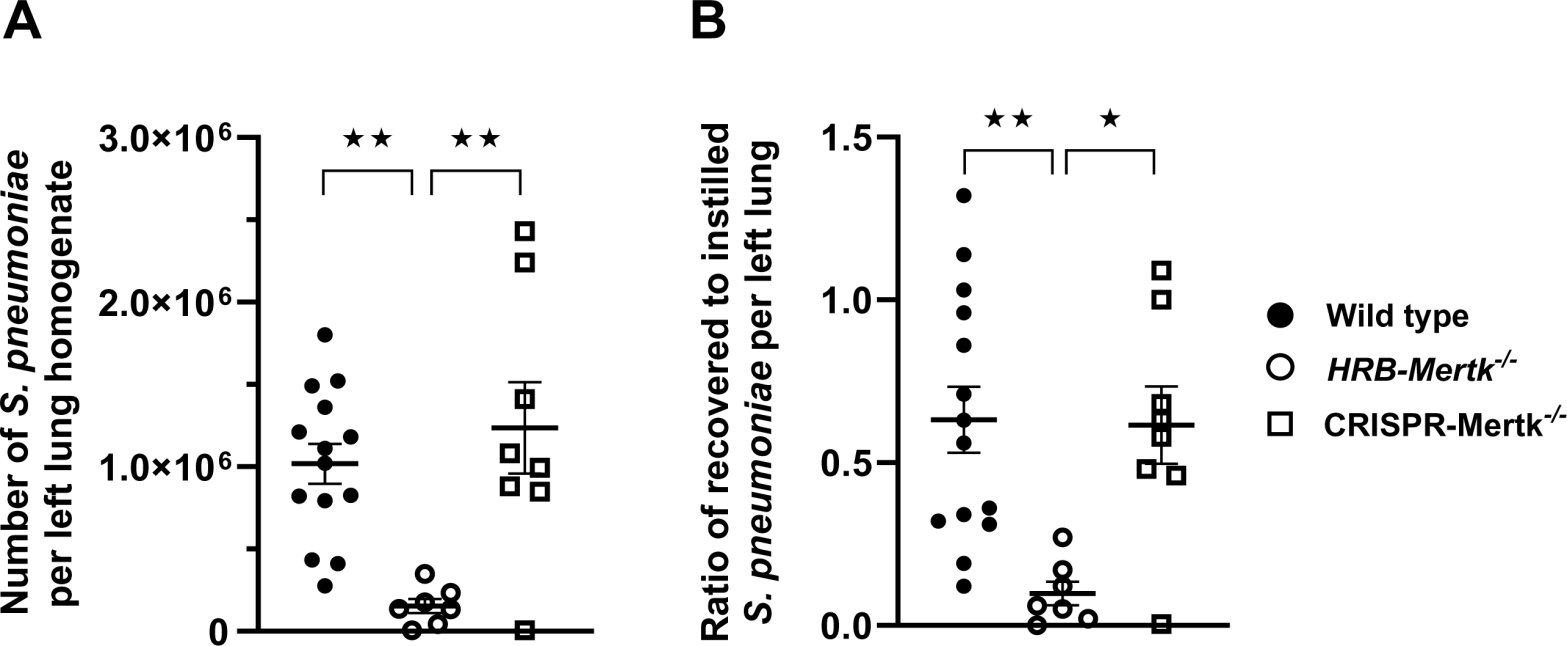
Enhanced bacterial clearance is not observed in CRISPR-*Mertk*^*-/-*^ mice. Bacterial clearance was assessed in HRB-*Mertk*^*-/-*^, CRISPR-*Mertk*^*-/-*^ and wild type male mice 24 hours post inoculation. The number of inoculated bacteria was based on body weight (3.43 ± 0.31x10^7^ CFU/mL). The (**A**) number of bacteria per left lung homogenate and (**B**) the ratio of recovered to instilled bacteria were determined, *n =* 7-14 per group. Data represent mean ±  SEM and were collected from two independent experiments. Statistically significant differences were determined by one-way ANOVA followed by Tukey’s multiple comparison test, ★*p* <  0.05, ★★ *p* < 0.01*.*

A recent report by Akalu and colleagues comparing murine models of Mertk deficiency showed that the *HRB-Mertk*^*-/-*^ mice carry multiple coincidental changes within their genome, and these changes alter the expression of many genes in a tissue and context-dependent manner [21]. Therefore, the phenotype of the HRB-*Mertk*^*-/-*^ mice is not solely due to deletion of Mertk. Using mice generated by one of our laboratories (HSE) through CRISPR technology that do not have any of these coincidental changes in their genome, we repeated studies of clearance comparing WT, HRB-*Mertk*^*-/-*^ and CRISPR-*Mertk*^*-/-*^ mice. The HRB-*Mertk*^*-/-*^ mice again showed the enhancement in clearance of *S. pneumoniae*, but the CRISPR-*Mertk*^*-/-*^ mice showed no defect in clearance (Fig 4). Thus, the very robust and significant enhancement in clearance is due either to a coincidentally altered gene (s) in the HRB-*Mertk*^*-/-*^ mice and/or to the function of these genomic alterations in lung tissue during pneumonia.

### Sex but not genotype impacts the number of myeloid cells in the lung of naïve and pneumonic HRB-*Mertk*^-/-^ and wild type mice

**Fig 5:**
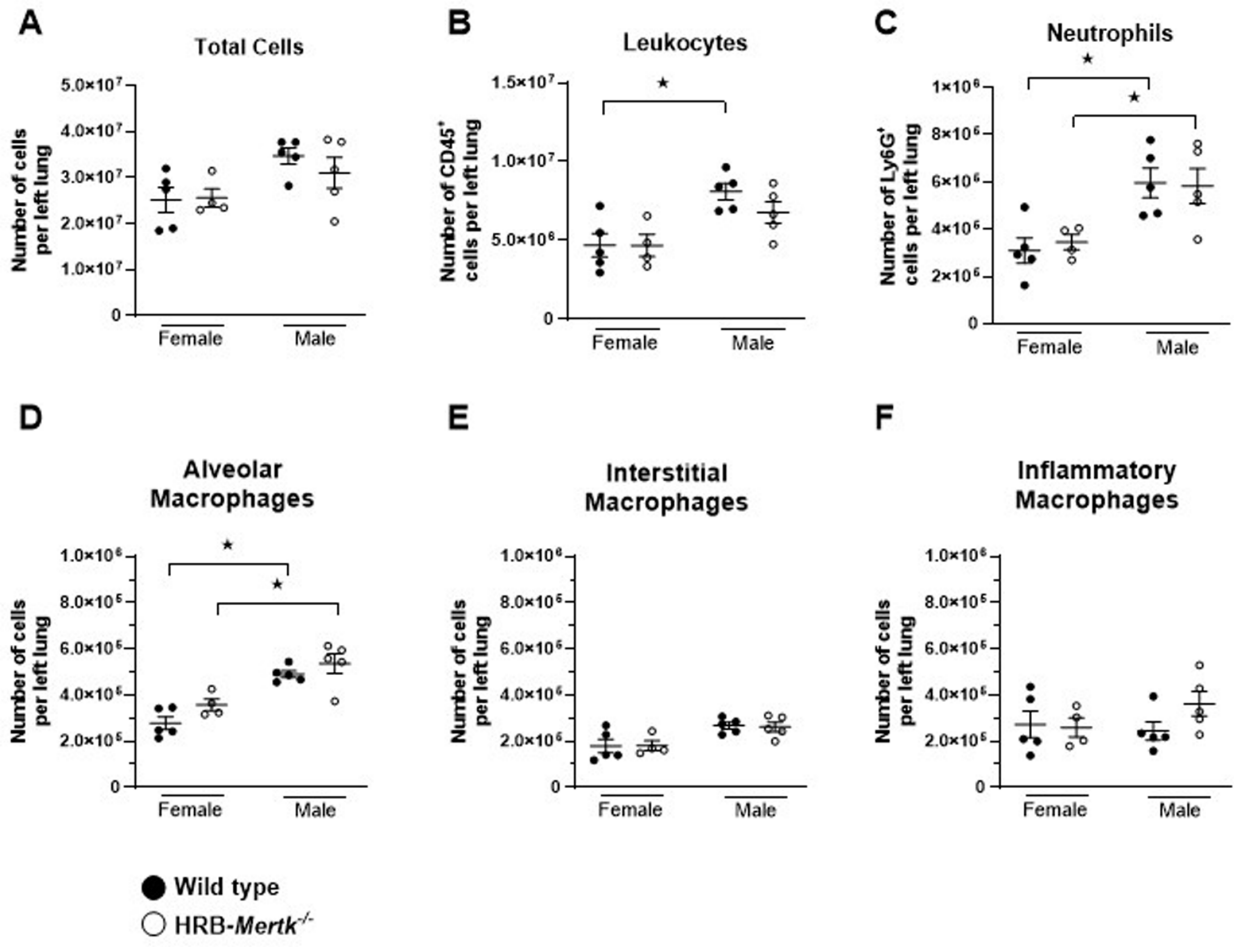
Naïve HRB-*Mertk*^*-/-*^ and wild type mice have similar numbers of immune cells in the left lung. The number of immune cells present in left lung homogenates of naïve HRB-*Mertk*^*-/-*^ and wild type mice was determined by flow cytometry. The (**A**) total cell count and the number of (**B**) leukocytes, (**C**) neutrophils, (**D**) alveolar macrophages, (**E**) interstitial macrophages, and (**F**) inflammatory macrophages were determined. Data were collected from 2 independent experiments and are expressed as the mean ±  SEM*.* Significant differences determined by one-way ANOVA followed by Tukey’s multiple comparison test. ★ *p* <  0.05.

Because the HRB-*Mertk*^*-/-*^ mice showed greater clearance at 24 hours post inoculation, we investigated their immune cell phenotype in naive and infected mice. The number of immune cells present in the left lung of naive HRB-*Mertk*^*-/-*^ and wild type mice were quantified by flow cytometry (n ≥  4 per group). The total number of cells present in left lung digests was similar in HRB-*Mertk*^*-/-*^ and wild type mice of both sexes (Fig 5A). There were fewer leukocytes in the lungs of wild type females compared to wild type males, and a similar trend was observed in HRB-*Mertk*^*-/-*^ mice (Fig 5B). There were fewer neutrophils in the lungs of female mice compared to male mice of both genotypes (Fig 5C). There were also fewer alveolar macrophages present in the lungs of female mice (Fig 5D). The number of either neutrophils or alveolar macrophages in naïve mice was not altered by genotype. The numbers of interstitial macrophages (Fig 5E), inflammatory macrophages (Fig 5F), and natural killer cells (S2A Fig in S1 File) were not affected by genotype or sex.

**Fig 6:**
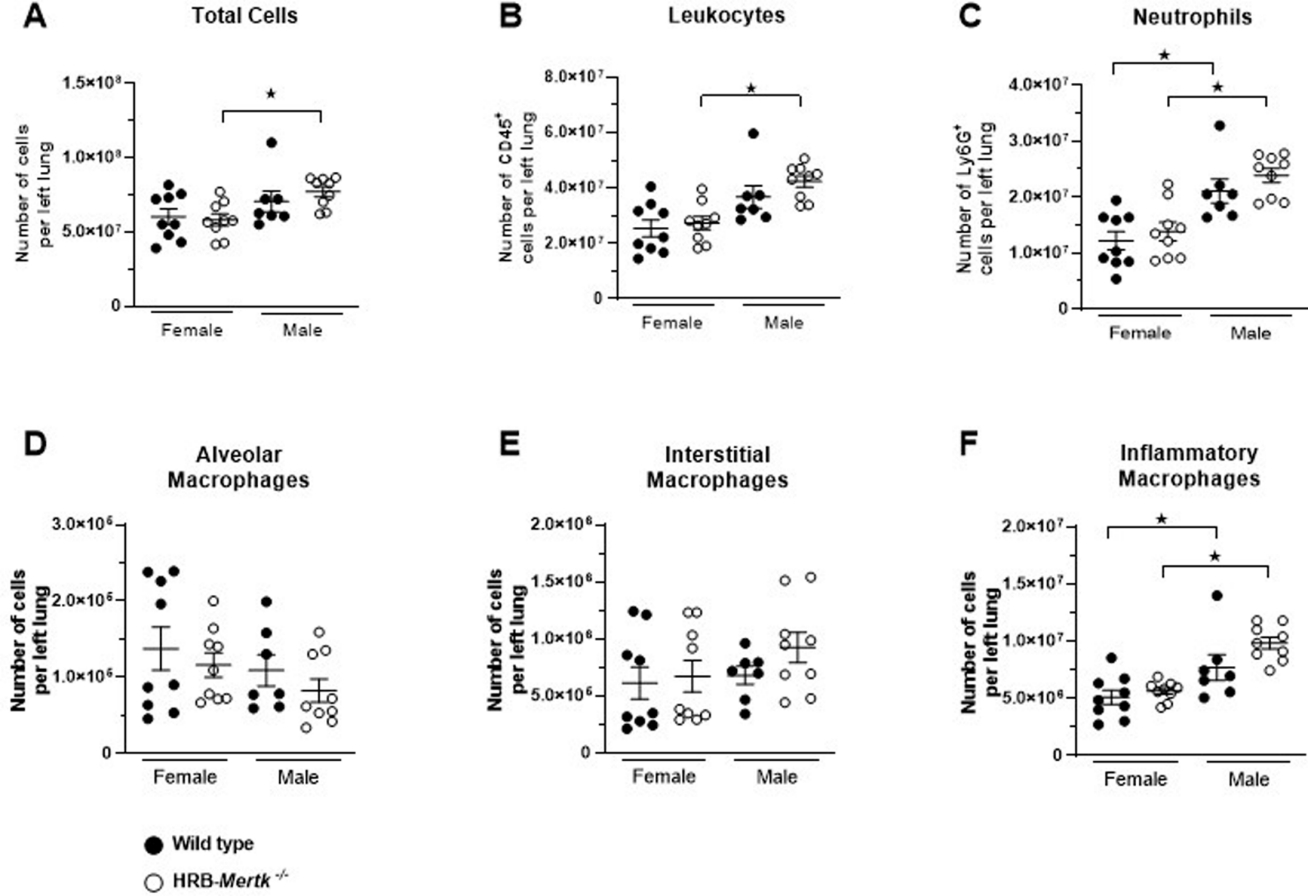
HRB-*Mertk*^*-/-*^ and wild type mice have a similar number of immune cells within the left lung 24 hours post-inoculation. The (**A**) total cell count of left lung digest obtained from *Mertk*^-/-^ and wild type mice 24 hours post-inoculation. The number of (**B**) leukocytes, (**C**) neutrophils, (**D**) alveolar macrophages, (**E**) interstitial macrophages, and (**F**) inflammatory macrophages was determined by flow cytometry. The number of inoculated bacteria was based on body weight (2.03 ± 0.49x10^7^ CFU/mL) Data were collected from two separate experiments and are expressed as mean ±  SEM. Significant differences determined by one-way ANOVA followed by Tukey’s multiple comparison test. ★ *p* <  0.05.

To determine if the improved bacterial clearance observed in HRB-*Mertk*^*-/-*^ mice was associated with altered recruitment of immune cells, left lung homogenates were prepared 24 hours post-inoculation and assessed by flow cytometry. There was no difference in the total number of cells present in left lung digests between the genotypes or sexes (Fig 6A). We identified a slight but significant increase in the number of CD45^ +^ cells in the lungs of HRB-*Mertk*^*-/-*^ males compared to HRB-*Mertk*^*-/-*^ females. A similar trend was observed in the number of CD45^ +^ cells between wild type male and female mice, but the difference was not statistically different (Fig 6B).

Neutrophils increased 5-8 fold by 24 hours post-inoculation when compared to naïve mice of both sexes and both genotypes (Fig 6C compared to Fig 5C). There were more neutrophils present in the lungs of male mice compared to female mice, but no difference between the genotypes (Fig 6C). The number of alveolar macrophages decreased 50-80% compared to naïve animals, but there were no differences identified between genotypes or sexes (Fig 6D). The number of interstitial macrophages increased between 2.5-3.5 fold compared to naïve animals, but there was no difference between genotypes or sexes (Fig 6E). The number of inflammatory macrophages increased during infection, between 18-27 fold. Significantly more inflammatory macrophages were recruited to the lungs of male mice compared to female mice, but there were no differences between genotypes (Fig 6F).

### HRB-*Mertk*^-/-^ mice have fewer neutrophils in airway lavage fluid 24 hours post-inoculation

**Fig 7:**
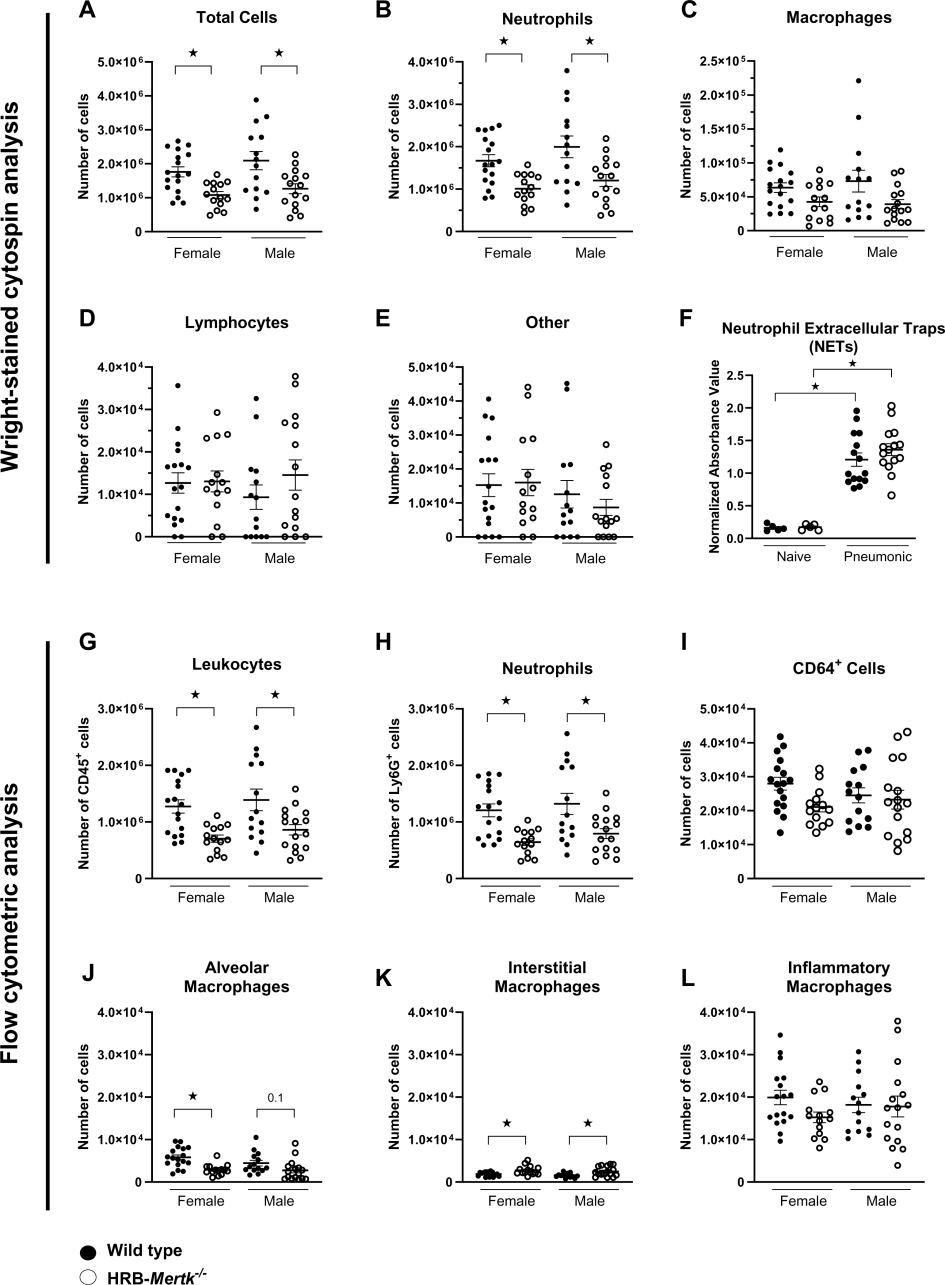
HRB-*Mertk*^*-/-*^ mice have fewer lavageable immune cells in the alveoli and airways 24 hours post-inoculation. Bronchoalveolar lavage (BAL) was performed on the left lung of *Mertk*^*-/-*^ and wild type mice 24 hours post-inoculation. The (**A**) total number of cells present in the BAL fluid was determined by hemocytometer. The number of (**B**) neutrophils, (**C**) macrophages, (**D**) lymphocytes and (**E**) other cell types was determined by differential cell analysis. BAL cells were also evaluated by flow cytometry. (**F**) Neutrophil extracellular traps (NETs) were quantified in the BAL fluid of naïve or pneumonic female mice 24 hours post inoculation using a modified sandwich ELISA that combined a capture antibody against myeloperoxidase and a detection antibody against DNA. Data represent mean normalized absorbance units (AU) ±  SEM. The number of (**G**) leukocytes, (**H**) neutrophils, (**I**) CD64^ +^ cells, (**J**) alveolar macrophages, (**K**) interstitial macrophages and (**L**) inflammatory macrophages was determined. The number of inoculated bacteria was based on body weight (2.37 ± 0.28x10^7^ CFU/mL). Data were collected from 4 independent experiments and are expressed as mean ±  SEM*.* Significant differences determined by one-way ANOVA followed by Tukey’s multiple comparisons test ★ *p* <  0.05.

To characterize immune cells present in the airway, we performed BAL of the left lung 24 hours post-inoculation. The total number of cells present in the BAL of HRB-*Mertk*^*-/-*^ mice was significantly lower than the number in wild type animals of both sexes (Fig 7A). The absolute number of immune cell subsets were determined using two approaches, Wright-stained cytospins and flow cytometry. Using cytospins, significantly fewer neutrophils were present in the BAL of HRB-*Mertk*^*-/-*^ compared to wild type mice of both sexes (Fig 7B). Neutrophils represented the majority of cells found in the BAL. The number of macrophages present in the BAL trended lower in HRB-*Mertk*^*-/-*^ compared to wild type mice (Fig 7C). The number of lymphocytes (Fig 7D) and other cell types (Fig 7E) were similar between genotypes.

Using BAL fluid obtained for the studies measuring mediators in the lung microenvironment, NET formation was evaluated using a sandwich ELISA to identify the myeloperoxidase that was associated with DNA. At 24 hours post inoculation, NET formation increased more than 5-fold in both genotypes, and there was no difference between genotypes in either naïve or pneumonic lungs (Fig 7F).

Flow cytometry was performed on the same BAL samples to determine the number of each macrophage subpopulation. Fewer CD45^ +^ cells were present in the BAL of HRB-*Mertk*^*-/-*^ compared to wild type mice (Fig 7G). This decrease was due primarily to fewer neutrophils, identified by Ly6G expression, in the BAL of HRB-*Mertk*^*-/-*^ compared to wild type mice (Fig 7H). The number of CD64^ +^ macrophages trended lower in HRB-*Mertk*^*-/-*^ females compared to wild type females, but not in males (Fig 7I). Evaluation of macrophage subpopulations identified significantly fewer alveolar macrophages in the BAL of HRB-*Mertk*^*-/-*^ females compared to wild type females (Fig 7J). There was a similar trend in HRB-*Mertk*^-/-^ *vs* wild type males. There were more interstitial macrophages present in the BAL of HRB-*Mertk*^*-/-*^ compared to wild type mice of both sexes (Fig 7K). The number of inflammatory macrophages present in the BAL was higher than the number of alveolar or interstitial macrophages but was not affected by either genotype or sex (Fig 7L).

### HRB-*Mertk*^-/-^ mice have increased IFNγ present in the BAL fluid, but an anti-IFNγ neutralizing antibody did not prevent the enhanced bacterial clearance

**Fig 8:**
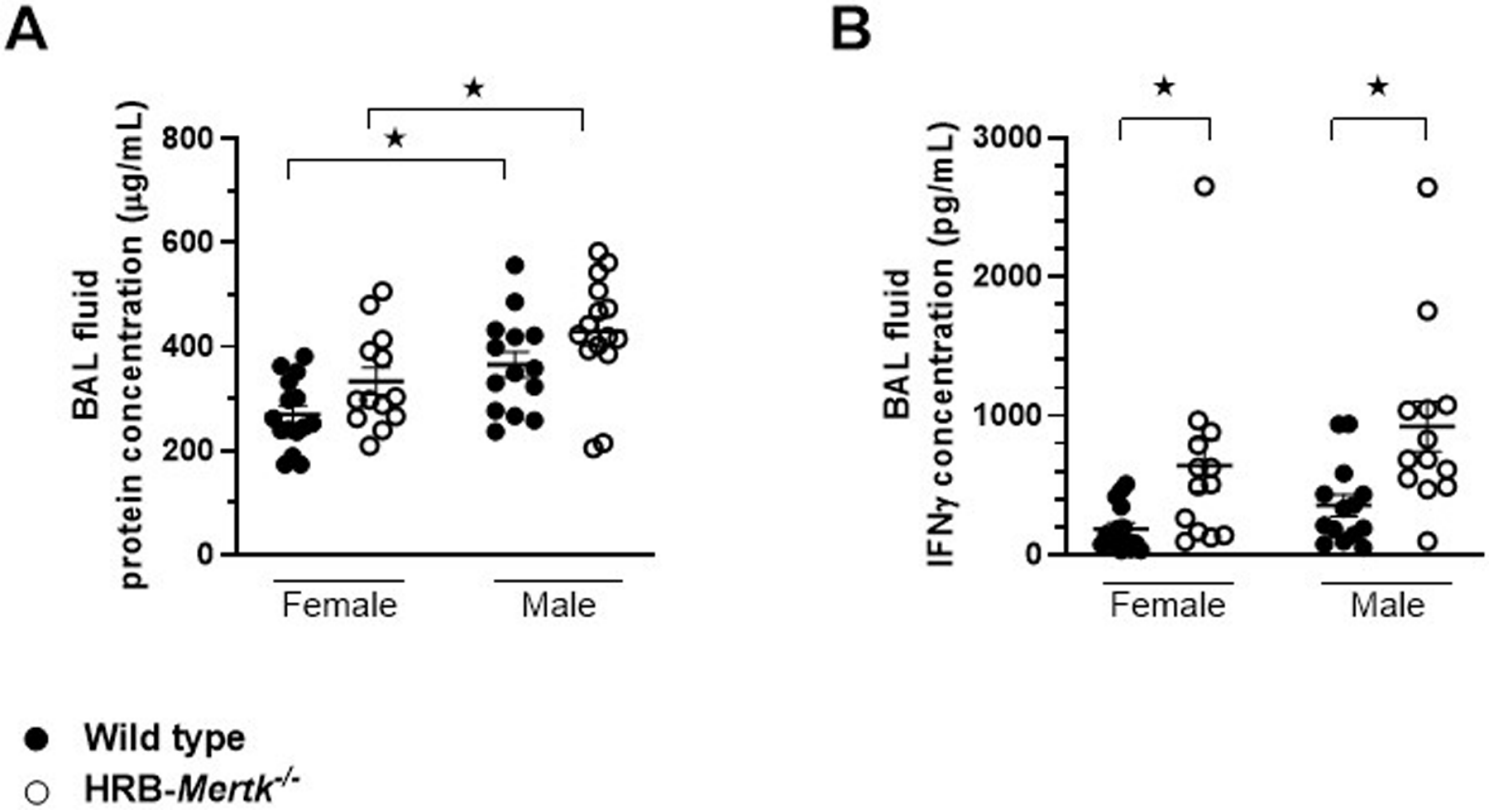
HRB-*Mertk*^-/-^ mice have increased IFN γ present in the BAL fluid 24 hours post inoculation. The concentration of IFNγ is greater in the BAL fluid of HRB-*Mertk*^*-/-*^ mice 24 post-inoculation. Quantification of (**A**) total protein concentration and (**B**) IFNγ concentration in the BAL fluid. The number of inoculated bacteria was based on body weight (2.37 ± 0.28x10^7^ CFU/mL), Data were collected from 4 independent experiments and are expressed as mean ±  SEM. Significant differences determined by one-way ANOVA followed by Fisher’s LSD test. ★ *p* <  0.05.

No differences in the total protein concentration of the BAL fluid were found between HRB-*Mertk*^*-/-*^ and sex-matched wild type animals, but males had higher concentrations compared to females of both genotypes (Fig 8A).

Concentrations of cytokines and other mediators in the BAL fluid of mice inoculated with *S. pneumoniae* for 24 hours were compared to that from two controls, naïve mice and mice instilled with PBS. There were no differences in any analyte between naïve HRB-*Mertk*^*-/-*^ and sex-matched wild type mice (Table 1). The instillation of PBS did not affect the concentration of any analyte in mice of the same sex and genotype (Table 1). In pneumonic mice, the concentration of all measured analytes, with the exception of IL-5, increased significantly compared to sex and genotype matched PBS controls (Table 1). Significant differences between genotypes that exceeded 1.5-fold in male mice with *S. pneumoniae* pneumonia include higher concentrations of IFNγ, IL-12 (p40), IL-17A and MCP-1 (CCL2) in HRB-*Mertk*^*-/-*^ compared to wild type males. Of these changes, only the increase in IFNγ concentration was also found in HRB-*Mertk*^*-/-*^ females compared to wild type females (p = 0.056). The concentration of TNFα was less in female HRB-*Mertk*^*-/-*^ mice and trended toward less in the male HRB-*Mertk*^*-/-*^ mice (Table 1). Because IFNγ may contribute to the clearance of *S. pneumoniae*, we confirmed this finding using an ELISA, identifying increased IFNγ concentrations in the BAL fluid of HRB-*Mertk*^*-/-*^ compared to wild type mice of both sexes (Fig 8B).

**Table 1:** Concentrations of cytokines and other mediators in the BAL fluid of mice inoculated with *S. pneumoniae* for 24 hours.

Analyte	Naïve				24-hour PBS				24-hour *S. pneumoniae*			
	Wild-type Female	*Mertk*^*-/-*^ Female	Wild-type Male	*Mertk*^*-/-*^ Male	Wild-type Female	*Mertk*^*-/-*^ Female	Wild-type Male	*Mertk*^*-/-*^ Male	Wild-type Female	*Mertk*^*-/-*^ Female	Wild-type Male	*Mertk*^*-/-*^ Male
**Eotaxin**	23.8 ± 6.0	17.6 ± 3.4	15.1 ± 2.3	16.4 ± 1.5	24.2 ± 2.4	22.3 ± 3.7	18.9 ± 2.5	15.1 ± 2.1	96.2 ± 9.2 ^‡^	123.0 ± 17.9 ^‡^	104.6 ± 10.6 ^‡^	140.0 ± 15.8 ^‡^
**G-CSF**	*n* = 5 < 6.0	*n* = 5 < 6.0	*n* = 5 < 6.0	*n* = 5 < 6.0	9.7, *n* = 1 (*n = *4 < 6.0)	*n = 5* < 6.0	*n = 5* < 6.0	*n = 5* < 6.0	3652.9 ± 379.5 ^‡^	3447.0 ± 233.5 ^‡^ ^◊^	3589.2 ± 397.8 ^‡^	4992.3 ± 458.1 ^‡^ ^●^
**GM-CSF**	5.5 ± 1.0, *n* = 2 (*n = *3 < 1.9)	7.9 ± 0.7, *n = *2 (*n = *3 < 1.9)	5.5 ± 1.0, *n = *2 (*n = *3 < 1.9)	6.5 ± 1.7, *n = *2 (*n = *3 < 1.9)	7.6 ± 1.5	4.7 ± 0.5	7.7 ± 1.9	6.8 ± 2.2	39.1 ± 2.7 ^‡^	35.0 ± 1.7 ^‡^	40.1 ± 1.6 ^‡^	41.7 ± 2.5 ^‡^
**IFN-γ**	4.2 ± 0.2	4.1 ± 0.4	4.4 ± 0.5	4.3 ± 0.2	5.2 ± 0.7	4.0 ± 0.6	4.4 ± 0.5	3.8 ± 0.7	47.7 ± 7.2 ^‡^	159.2 ± 51.6 ^‡^	49.5 ± 11.2 ^‡^	362.5 ± 123.3 ^‡^ ^●^
**IL-1α**	5.1 ± 0.8	3.3 ± 0.5	2.7 ± 0.5	2.9 ± 0.4	4.0 ± 0.3	3.1 ± 0.5	3.0 ± 0.5	2.7 ± 0.3	50.7 ± 3.8 ^‡^	50.5 ± 4.9 ^‡^	47.8 ± 3.7 ^‡^	63.3 ± 7.9 ^‡^
**IL-1β**	*n* = 5 < 1.4	*n* = 5 < 1.4	*n* = 5 < 1.4	*n* = 5 < 1.4	*n* = 5 < 1.4	*n* = 5 < 1.4	*n* = 5 < 1.4	*n* = 5 < 1.4	33.8 ± 2.6 ^‡^ ^◊^	41.5 ± 3.1 ^‡^ ^◊^	44.7 ± 3.4 ^‡^	55.7 ± 3.4 ^‡^ ^●^
**IL-2**	3.1 ± 0.6	3.1 ± 0.7	3.9 ± 1.1, *n = *4 (*n = *1 < 1.3)	3.5 ± 0.6	3.6 ± 0.8	3.5 ± 0.7, *n* = 4 (*n* = 1 < 1.3)	3.7 ± 0.7	3.4 ± 0.2	6.2 ± 0.3 ^‡^	6.1 ± 0.2 ^‡^ ^◊^	5.5 ± 0.3 ^‡^	7.0 ± 0.4 ^‡^ ^●^
**IL-3**	*n* = 5 < 0.7	*n* = 5 < 0.7	*n* = 5 < 0.7	*n* = 5 < 0.7	*n* = 5 < 0.7	*n* = 5 < 0.7	*n* = 5 < 0.7	*n* = 5 < 0.7	1.1 ± 0.1 ^‡^ ^◊^	1.1 ± 0.1 ^‡^	0.9 ± 0.1 ^‡^	1.2 ± 0.1 ^‡^ ^●^
**IL-4**	0.6 ± 0.1, *n* = 3 (*n = *2 < 0.5)	0.55, *n* = 1 (*n = *4 < 0.5)	*n* = 5 < 0.5	0.7 ± 0.1, *n* = 2 (*n = *3 < 0.5)	0.8 ± , *n* = 1 (*n = *4 < 0.5)	(*n = *5 < 0.5)	1.7, *n* = 1 (*n = *4 < 0.5)	1.0, *n* = 1 (*n = *4 < 0.5)	2.0 ± 0.2 ^‡^ ^◊^	3.9 ± 0.6 ^‡^ ^●^	4.7 ± 0.3 ^‡^	4.2 ± 0.4 ^‡^
**IL-5**	*n* = 5 < 3.5	*n* = 5 < 3.5	*n* = 5 < 3.5	*n* = 5 < 3.5	*n* = 5 < 3.5	*n* = 5 < 3.5	*n* = 5 < 3.5	*n* = 5 < 3.5	5.4 ± 0.9, *n* = 6 (*n* = 4 < 3.5)	4.1 ± 0.3, *n* = 3 (*n* = 6 < 3.5)	3.7, *n* = 1 (*n* = 9 < 3.5)	4.9 ± 0.5, *n* = 4 (*n* = 6 < 3.5)
**IL-6**	*n* = 5 < 2.6	*n* = 5 < 2.6	*n* = 5 < 2.6	*n* = 5 < 2.6	*n* = 5 < 2.6	*n* = 5 < 2.6	*n* = 5 < 2.6	*n* = 5 < 2.6	589.8 ± 46.9 ^‡^	516.2 ± 36.4 ^‡^ ^◊^	493.0 ± 35.7 ^‡^	728.7 ± 69.8 ^‡^ ^●^
**IL-9**	3.6 ± 0.4, *n* = 2 (*n = *3 < 2.7)	3.2 ± 0.2, *n* = 2 (*n = *3 < 2.7)	3.2, *n* = 1 (*n = *4 < 2.7)	3.5, *n* = 1 (*n = *4 < 2.7)	3.8, *n* = 1 (*n = *4 < 2.7)	(*n = *5 < 2.7)	3.2, *n* = 1 (*n = *4 < 2.7)	2.8, *n* = 1 (*n = *4 < 2.7)	11.2 ± 0.8 ^‡^	15.9 ± 5.6 ^‡^	8.3 ± 0.3 ^‡^	10.5 ± 1.1 ^‡^
**IL-10**	*n* = 5 < 14.5	*n* = 5 < 14.5	*n* = 5 < 14.5	*n* = 5 < 14.5	*n* = 5 < 14.5	*n* = 5 < 14.5	*n* = 5 < 14.5	*n* = 5 < 14.5	37.9 ± 1.8 ^‡^	40.1 ± 1.6 ^‡^	36.1 ± 1.1 ^‡^	37.0 ± 1.9 ^‡^
**IL-12 (p40)**	78.4 ± 13.0 ^◊^	71.3 ± 16.2	37.4 ± 1.8	37.6 ± 3.1	48.8 ± 3.8 ^◊^	57.2 ± 5.3 ^◊^	27.1 ± 1.3	29.6 ± 4.2	1428.4 ± 200.6 ^‡^ ^◊^	1103.4 ± 284.7 ^‡^	496.3 ± 31.3 ^‡^	1136.8 ± 201.2 ^‡^ ^●^
**IL-12 (p70)**	13.0 ± 1.5, *n* = 4 (*n = *1 < 8.0)	10.1 ± 0.8, *n* = 3 (*n = *2 < 8.0)	10.0 ± 0.8, *n* = 2 (*n* = 3 < 8.0)	10.0 ± 1.4, *n* = 2 (*n = *3 < 8.0)	12.0 ± 1.0, *n* = 4 (*n* = 1 < 8.0)	11.3 ± 1.4, *n* = 3 (*n* = 2 < 8.0)	11.9, *n* = 1 (*n* = 4 < 8.0)	10.0 ± 0.8, *n* = 2 (*n* = 3 < 8.0)	267.3 ± 36.4 ^‡^	273.3 ± 32.7 ^‡^	202.7 ± 16.9 ^‡^	282.0 ± 43.4 ^‡^
**IL-13**	15.8 ± 1.7	19.8 ± 1.4, *n* = 4 (*n = *1 < 12.9)	18.9 ± 2.4, *n* = 4 (*n = *1 < 12.9)	18.3 ± 0.9	18.5 ± 2.8, *n* = 4 (*n* = 1 < 12.9)	17.8 ± 2.1, *n* = 3 (*n* = 2 < 12.9)	14.0 ± 0.9, *n* = 3 (*n* = 2 < 12.9)	16.7 ± 1.2, *n* = 4 (*n* = 1 < 12.9)	41.2 ± 1.4 ^‡^	44.6 ± 1.4 ^‡^ ^◊^	43.0 ± 1.3 ^‡^	52.9 ± 1.9 ^‡^ ^●^
**IL-17A**	3.6 ± 0.9 ^◊^	2.7 ± 0.4	1.8 ± 0.4	2.2 ± 0.3	3.7 ± 0.2 ^◊^	3.0 ± 0.2	2.3 ± 0.5	2.8 ± 0.2	63.5 ± 12.2 ^‡^	61.4 ± 12.4 ^‡^ ^◊^	30.5 ± 3.3 ^‡^	135.1 ± 40.4 ^‡^ ^●^
**KC**	12.3 ± 2.1	15.3 ± 2.5	8.8 ± 0.9	12.0 ± 2.5	14.8 ± 1.5	14.5 ± 0.9	13.6 ± 1.3	13.4 ± 1.6	188.0 ± 18.9 ^‡^	256.9 ± 34.3 ^‡^	134.6 ± 8.3 ^‡^	257.7 ± 40.5 ^‡^ ^●^
**MCP-1**	43.7 ± 2.6, *n = *5	47.5 ± 4.6, *n = *4 (*n* = 1 < 14.1)	35.5 ± 4.5	47.0 ± 6.8	52.4 ± 9.4	47.8 ± 4.8	52.3 ± 4.5	50.5 ± 3.1	1588.5 ± 206.4 ^‡^ ^◊^	1272.3 ± 186.6 ^‡^	859.9 ± 85.6 ^‡^	1581.4 ± 275.3 ^‡^ ^●^
**MIP-1α**	*n* = 5 < 0.3	0.36 ± 0.1, *n* = 3 (*n* = 2 < 0.3)	*n* = 5 < 0.3	*n* = 5 < 0.3	0.4 ± 0.1	0.4 ± 0.1	0.4 ± 0.1, *n* = 3 (*n* = 2 < 0.3)	0.5 ± 0.1, *n* = 4 (*n* = 1 < 0.3)	402.8 ± 38.2 ^‡^	315.2 ± 34.2 ^‡^	389.0 ± 42.0 ^‡^	429.4 ± 58.6 ^‡^
**MIP-1β**	*n* = 5 < 7.4	*n* = 5 < 7.4	*n* = 5 < 7.4	*n* = 5 < 7.4	*n* = 5 < 7.4	*n* = 5 < 7.4	*n* = 5 < 7.4	*n* = 5 < 7.4	3180.2 ± 439.2 ^‡^, *n* = 6 (*n = *4 > 9207.1)	3038.7 ± 375.1 ^‡^, *n* = 7 (*n = *2 > 9207.1)	3106.9 ± 309.1 ^‡^, *n* = 9 (*n = *1 > 9207.1)	4021.8 ± 815.2 ^‡^, *n* = 7 (*n = *3 > 9207.1)
**RANTES**	5.0 ± 0.2, *n* = 4 (*n = *1 < 3.8)	5.0 ± 0.4, *n* = 4 (*n = *1 < 3.8)	4.6 ± 0.5, *n* = 3 (*n = *2 < 3.8)	5.1 ± 0.2, *n* = 3 (*n = *2 < 3.8)	4.6 ± 0.3, *n* = 3 (*n = *2 < 3.8)	4.5, *n* = 1 (*n = *4 < 3.8)	4.4 ± 0.3, *n* = 2 (*n = *3 < 3.8)	5.4, *n* = 1 (*n = *4 < 3.8)	1209.5 ± 187.2 ^‡^	1248.9 ± 169.8 ^‡^	1584.5 ± 254.1 ^‡^	1608.7 ± 160.5 ^‡^
**TNF-α**	*n* = 5 < 3.6	*n* = 5 < 3.6	*n* = 5 < 3.6	*n* = 5 < 3.6	*n* = 5 < 3.6	*n* = 5 < 3.6	*n* = 5 < 3.6	*n* = 5 < 3.6	2142.9 ± 506.0 ^‡^ ^◊^	907.6 ± 309.3 ^‡^ ^●^	434.5 ± 148.3 ^‡^	1252.9 ± 343.5 ^‡^

Cell free BAL fluid from naïve mice (n = 5/group), those instilled with phosphate buffered saline (PBS, n = 5/group), or S. pneumoniae suspension (n = 9-10/group) for 24 hours were analyzed using a multi-plex cytokine assay. Differences were determined by one-way ANOVA followed by uncorrected Fisher’s LSD test on the following preselected comparisons: ☐p < 0.05 naive vs saline, same sex and genotype;

‡ p < 0.05 saline vs S. pneumoniae, same sex and genotype;

◊ p < 0.05 female vs male mice, same genotype and treatment;

● p < 0.05 Mertk^-/-^ vs wild-type, same sex and treatment. The number of samples which did not fall within the working range of the assay for a specific analyte indicated where appropriate.

**Fig 9:**
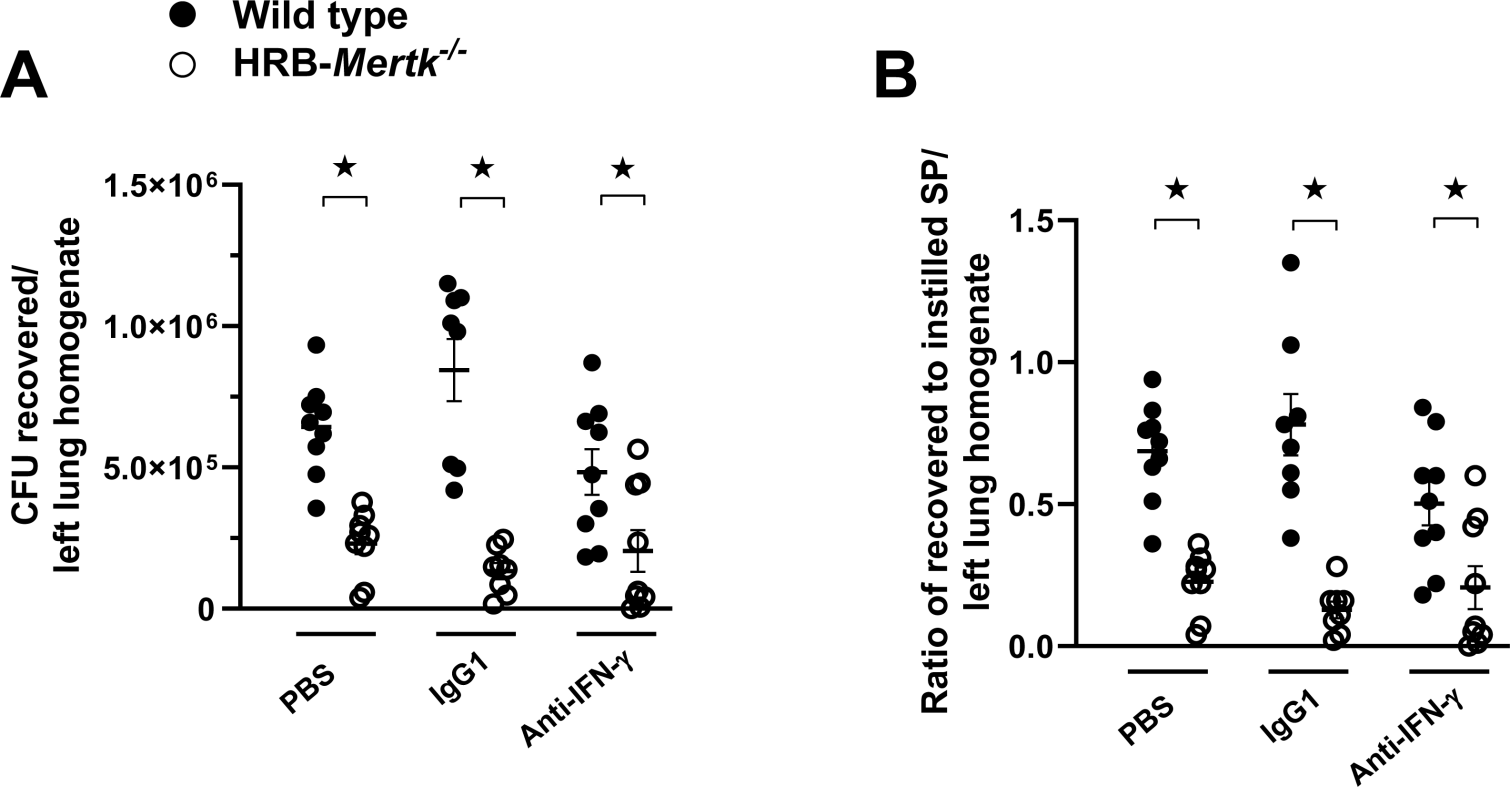
Enhanced bacterial clearance in HRB-*Mertk*^*-/-*^ is not altered by neutralization of IFNγ. HRB-*Mertk*^*-/-*^ and wild type received blocking anti-murine IFNγ antibody intraperitoneally 1 hour prior to inoculation and the same antibody intratracheally combined with the inoculum. Two control groups, PBS or nonimmune IgG, were included in each of two independent experiments. The (**A**) number of colony forming units (CFU) per left lung homogenate and (**B**) ratio of recovered to instilled *S. pneumoniae* (SP) were determined 24 hours post-inoculation. The number of inoculated bacteria was based on body weight (2.40 ± 0.16x10^7^ CFU/mL). Data represent mean ±  SEM*.* Significant differences determined by one-way ANOVA followed by Tukey’s multiple comparison test. ★ *p* <  0.05.

We postulated that the increased concentration of IFNγ contributes to the enhanced clearance of *S. pneumoniae* in HRB-*Mertk*^*-/-*^ mice. To test this hypothesis, wild type and HRB-*Mertk*^*-/-*^ received a blocking anti-murine IFNγ antibody intraperitoneally 1 hour before inoculation and the same antibody intratracheally combined with the inoculum. Bacterial clearance measured 24 hours post inoculation showed the same enhancement of clearance in HRB-*Mertk*^*-/-*^ compared to wild type mice receiving anti-IFNγ antibody as was seen in both controls (mice given either PBS or nonimmune IgG instead of anti-murine IFNγ antibody, Fig 9). These data suggest that the increased IFNγ present in HRB-*Mertk*^*-/-*^ mice does not, alone, account for the enhancement of clearance.

### The transcriptome of naïve alveolar macrophages is altered in HRB-*Mertk*^-/-^ mice

Because the enhancement of clearance in HRB-*Mertk*^*-/-*^ mice occurred rapidly, within 24 hours, and the alveolar macrophages were the site of most Mertk expression amongst macrophages (Fig 1), the transcriptomes of naïve alveolar macrophages were compared. Alveolar macrophages were collected from naïve female and male HRB-*Mertk*^*-/-*^ and wild type mice using BAL of both lungs. There were no differences in the number of cells retrieved between sex or genotype, and all samples consisted of more than 98% macrophages as determined by differential cell counts from cytospins (S3 Fig in S1 File). Total RNA was isolated and RNA sequencing was performed. A differentially expressed gene (DEG) was defined as having an average log2 expression amongst all samples >  0, an FDR adjusted p value <  0.05 and a fold change greater than ±  1.5.

**Fig 10:**
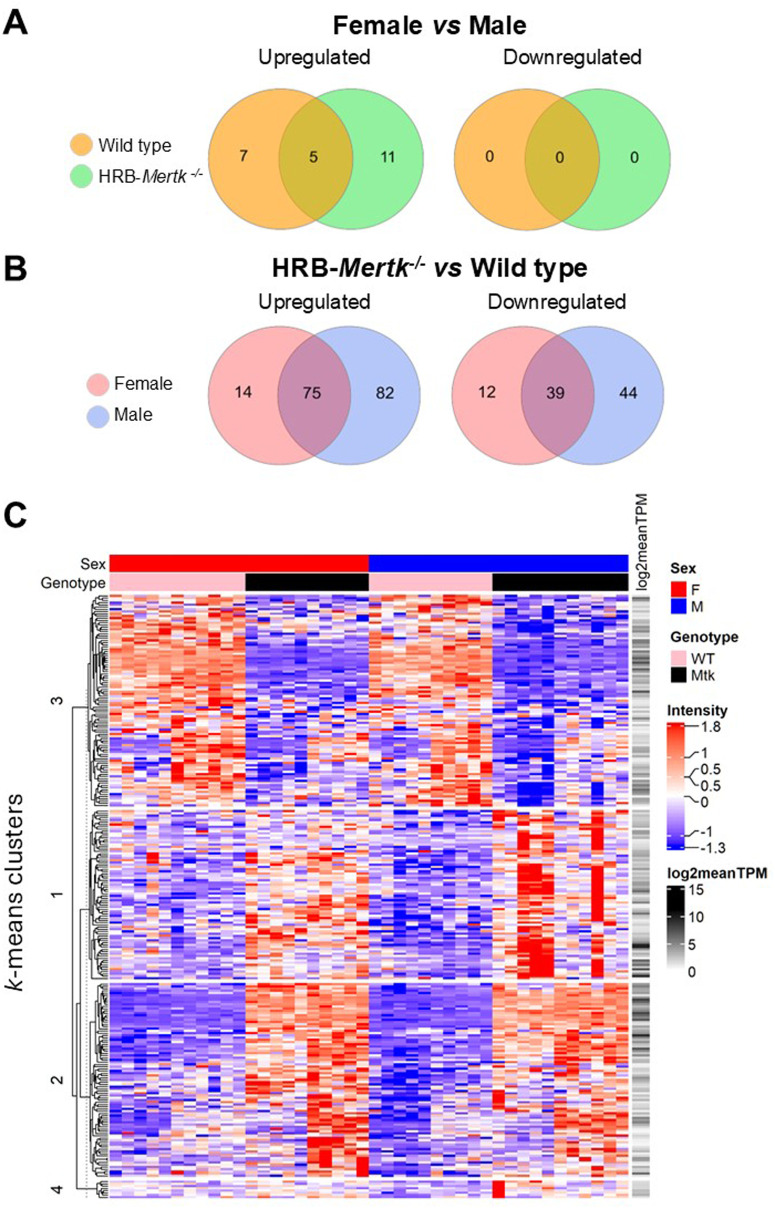
Alveolar macrophages of naive HRB-*Mertk*^*-/-*^ mice have an altered transcriptome. Transcriptomic analysis was performed on naïve alveolar macrophages isolated from HRB-*Mertk*^*-/-*^ or wild type mice (*n = *10-11 per sex and genotype). As described in the methods, a differentially expressed gene (DEG) was defined as average expression >  0, FDR < 0.05, and fold change>  ± 1.5. Venn diagrams representing (A) the number of DEGs identified in female *vs* male mice of the same genotype or (B) the number of DEGs identified in HRB-*Mertk*^*-/-*^ *vs* wild type alveolar macrophages. **(C)** Heatmap depicting hierarchical clustering and *k-*means analysis using Euclidean distance metric of DEGs. The dendrogram (left) indicates clustering across transcripts (log2 normalized intensity) and each column represents an individual animal of the indicated sex and genotype (top).

We evaluated the impact of sex on the transcriptome of alveolar macrophages isolated from mice of the same genotype. Comparing female to male mice, 12 DEGs were upregulated in wild type females, and 16 DEGs were upregulated in HRB- females (S1 Tab in S2 File). Of these, 5 were common to both genotypes (*Eif2s3x, Gm2223, Kdm6a, Ndrg1* and *Tlr8*), four of which are located on the X chromosome. No DEGs were downregulated in females compared to males (Fig 10A).

Comparison of the transcriptomes of naïve HRB-*Mertk*^*-/-*^ and wild type alveolar macrophages revealed 140 DEGs in HRB-*Mertk*^*-/-*^ *vs* wild type females (S2 Tab in S2 File) and 240 DEGs in HRB-*Mertk*^*-/-*^ *vs* wild type males (S3 Tab in S2 File). Of the total DEGs identified between genotypes, 114 were changed similarly in both sexes, 26 were changed uniquely in female mice, and 126 were changed uniquely in male mice (Fig 10B). Known functions of these DEGs included cellular metabolic processes, cytoskeletal and membrane alterations, gene expression and transcriptional regulation, and the generation of or response to inflammatory mediators. Functional enrichment analysis (fgsea) performed using all expressed genes identified in both male and female comparisons revealed 11 GO terms that were significantly enriched in HRB-*Mertk*^*-/-*^ alveolar macrophages, which described leukocyte migration, the response to reactive oxygen species, leukocyte activation and regulation of the inflammatory response (S4 Tab in S2 File).

Hierarchical clustering and subsequent *k*-means analysis of all DEGs revealed 4 clusters (Fig 10C). Gene list over-representation using DEGs identified within each cluster revealed that genes in cluster 1, which are upregulated in HRB-*Mertk*^*-/-*^ alveolar macrophages, are associated with GO terms such as leukocyte adhesion and aggregation, cellular motility, and regulation of inflammatory response (S5 Tab in S2 File). Genes in cluster 4, also upregulated in HRB-*Mertk*^*-/-*^ alveolar macrophages, are associated with the response to bacteria, response to interferon-beta, and regulation of interleukin-1 (S5 Tab in S2 File). No GO terms were significantly enriched in clusters 2 or 3.

### The transcriptome of alveolar macrophages from HRS-*Mertk*^-/-^ mice is altered following exposure to *S. pneumoniae ex vivo*

To further evaluate the effect of *S. pneumoniae* on the transcriptome of alveolar macrophages, BAL macrophages were studied *ex vivo.* BAL macrophages were lavaged from naïve HRB-*Mertk*^*-/-*^ or wild type male mice and cultured for 24 hours, followed by exposure to *S. pneumoniae* or medium (unstimulated) for 4 hours. Following RNA sequencing, analysis compared alveolar macrophages exposed to either no stimulus or *S. pneumoniae* for each genotype, and then between genotypes.

Comparison of unstimulated alveolar macrophages identified 22 upregulated and 15 downregulated DEGs in HRB-*Mertk*^*-/-*^ *vs* wild type cells (S6 Tab in S2 File). Of these, 16 upregulated (*Gm18562, Pcna-ps2, Eif3j2, Gatm, Bend4, Plcb1, Steap3, Atp10d, Csde1, Ppip5k1, Tmem181b-ps, Nras, Sike1, Cdkl2, Gpr137b,* and *Cds1*) and 13 downregulated (*Crlf2*, *H2bc4, Eif3j1, Mertk, Idua, Mctp1, Bfsp1, Stard9, Spint1, Bub1b, Ero1b, Gpr137b-ps,* and *Stbd1*) DEGs were similarly identified in freshly isolated naïve HRB-*Mertk*^*-/-*^ alveolar macrophages described above. No GO terms or Reactome pathways were identified by functional enrichment analysis.

Comparison of *S. pneumoniae*-stimulated alveolar macrophages identified 17 upregulated and 18 downregulated DEGs in HRB-*Mertk*^*-/-*^ *vs* wild type cells (S7 Tab in S2 File). Functional enrichment analysis identified 10 GO terms (S8 Tab in S2 File) significantly enriched in stimulated HRB-*Mertk*^*-/-*^ alveolar macrophages. GO terms associated with these DEGs describe cellular adhesion, cell motility and migration, and lipid kinase activity (S8 Tab in S2 File). No Reactome pathways were identified.

**Fig 11:**
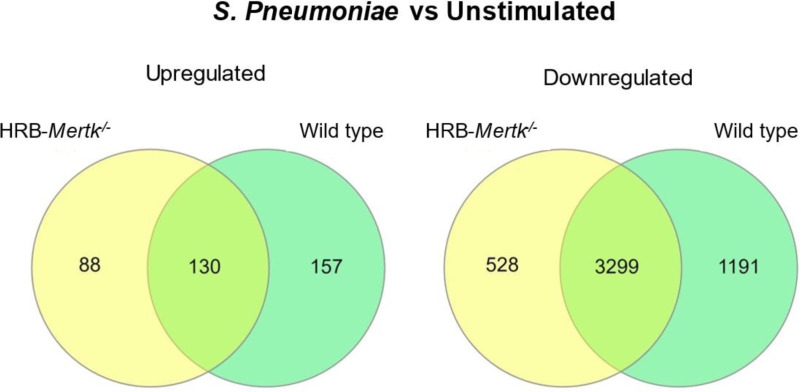
Alveolar macrophages from HRB-*Mertk*^*-/-*^ mice exhibit differential gene expression when stimulated with *S. pneumoniae.* Transcriptomic analysis was performed on *ex vivo* alveolar macrophages isolated from HRB-*Mertk*^*-/-*^ or wild type mice. As described in the methods, a differentially expressed gene (DEG) was defined as average expression >  0, FDR < 0.05, and fold change>  ± 1.5. Venn diagrams representing the number of upregulated or downregulated DEGs identified in *S. pneumoniae vs* unstimulated alveolar macrophages.

The number of DEGs that were induced by *S. pneumoniae* was determined by comparing stimulated to unstimulated macrophages of the same genotype. 4,045 DEGs were identified in stimulated HRB-*Mertk*^*-/-*^ cells (S9 Tab in S2 File) and 4,777 DEGs in wild type cells (S10 Tab in S2 File). Of these, 616 were uniquely expressed in HRB-*Mertk*^*-/-*^ cells and 1,348 were uniquely expressed in wild type cells (Fig 11). Functional enrichment analysis identified 515 GO terms enriched in HRB-*Mertk*^-/-^ cells (S11 Tab in S2 File) and 176 terms enriched in wild type cells (S12 Tab in S2 File) following *S. pneumoniae* exposure*;* 385 were unique to HRB-*Mertk*^*-/-*^ (S11 Tab in S2 File, rows 134-528) and 46 to wild type (S12 Tab in S2 File, rows 134-189). Similarly, 81 Reactome pathways were identified in HRB-*Mertk*^*-/-*^ cells (S13 Tab in S2 File) and 66 in wild type cells (S14 Tab in S2 File); 40 were unique to stimulated HRB-*Mertk*^*-/-*^ cells (S13 Tab in S2 File, rows 45-94) and 25 were unique to wild type cells (S14 Tab in S2 File, rows 45-79).

These GO terms or Reactome pathways uniquely identified in either genotype were manually grouped into seven categories describing DNA replication and transcriptional regulation, T cell/NK cell/B cell mediated immune processes, cellular response to hypoxia and regulation of reactive oxygen species and nitric oxide metabolic processes, biosynthetic/catabolic processes, cellular metabolism, cellular motility and migration, responses to inflammatory stimuli/signaling, and production of inflammatory mediators. The pathways that were enriched in HRB-*Mertk*^*-/-*^ macrophages included DNA replication and transcriptional regulation, T cell mediated immunity, proliferation and differentiation, NK cell- and B cell-mediated immune processes, cellular response to hypoxia and regulation of reactive oxygen species and nitric oxide metabolic processes, cellular biosynthetic/catabolic pathways, cellular motility and migration, responses to inflammatory stimuli/signaling, and production of inflammatory mediators. Transcriptomic pathways unique to wild type macrophages included response to endoplasmic reticulum stress and processes involved in increasing the rate of mitochondrial respiration.

Protein coding transcripts that show > 2-fold difference in expression between genotypes were identified. Amongst the DEGs induced by *S. pneumoniae* common to both genotypes, 31 show > 2-fold difference in HRB-*Mertk*^*-/-*^ cells and 44 in wild type cells. The analysis also identified 79 protein coding transcripts uniquely expressed in stimulated HRB-*Mertk*^*-/-*^ cells and 189 unique to wild type cells with > 2-fold difference in fold changes. Individual genes and fold changes identified are listed in S15 Tab in S2 File. Of those genes similarly identified in both genotypes, HRB-*Mertk*^*-/-*^ alveolar macrophages showed increased expression of Il1b (6-fold), Tnf (3-fold), Cxcl1 (8-fold), Cxcl2 (10-fold), and Cxcl3 (5-fold) compared to wild type cells. Of the 13 DEGs that had > 3-fold differences in wild type cells, all were decreased compared to stimulated HRB-*Mertk*^*-/-*^ macrophages except for *Bpifa1. Bpifa1* expression was increased 3.3-fold in stimulated wild type macrophages, and the gene product (SPLUNC1) has a protective role in bacterial infections.

Taken together, these data suggest that the alterations in the genome of the HRB-*Mertk*^*-/-*^ *mice* affect the transcriptome of infected alveolar macrophages in ways that are likely to upregulate host defense pathways, adhesion, motility, migration and locomotion, potentially enhancing clearance. Importantly, GO gene sets associated with phagocytosis were enriched in alveolar macrophages exposed to *S. pneumoniae* compared to uninfected macrophages in both genotypes, but there was no difference in the enrichment between HRB-*Mertk*^*-/-*^ and wild type cells.

### The left lung transcriptome of HRB-*Mertk*^-/-^ mice is altered in both naïve animals and during *S. pneumoniae* infection

To evaluate the effects of Mertk deficiency on the lung transcriptome, bulk RNA-sequencing was performed on left lung tissue of naive male mice and on pneumonic lung tissue at 24 hours post *S. pneumoniae* inoculation. As before, a DEG was defined as having an average log2 expression across all samples >  0, a FDR < 0.05 and a fold change>  ±  1.5. Naïve lung tissue of either genotype were compared. The effect of *S. pneumoniae* was evaluated using two approaches. First, pneumonic lung tissue from HRB-*Mertk*^*-/-*^ and wild type mice were compared directly. Second, pneumonic tissue was compared to naïve tissue for each genotype. Then, those DEGs induced by *S. pneumoniae* in HRB-*Mertk*^*-/-*^ or wild type mice were compared between genotypes. The results of each analysis are described below.

*Comparison of naïve lung tissue between genotypes* There were 42 upregulated and 41 downregulated DEGs in the left lung of HRB-*Mertk*^*-/-*^ *vs* wild type mice (S16 Tab in S2 File). No GO terms or Reactome pathways were identified by functional enrichment analysis of DEGs. Interestingly, 17 upregulated and 20 downregulated DEGs identified in naïve HRB-*Mertk*^*-/-*^ lung tissue were also identified in naive HRB-*Mertk*^*-/-*^ alveolar macrophages to have a similar change in expression. *Sptbn5* was also identified as changed, but was upregulated in HRB-*Mertk*^*-/-*^ alveolar macrophages and downregulated in HRB-*Mertk*^*-/-*^ lung tissue. These genes and their respective fold changes in naïve alveolar macrophages and left lung tissue are listed in Table 2. No GO terms or Reactome pathways were significantly enriched in naïve lung tissue.

**Table 2:** The fold change of DEGs that were identified in both HRB-*Mertk*^*-/-*^ alveolar macrophages and naïve left lung tissue.

	Naïve Alveolar Macrophages		Naïve Left Lung
Gene Symbol	Female *HRB-Mertk*^*-/-*^ *vs* wild type	Male *HRB-Mertk*^*-/-*^ *vs* wild type	Male *HRB-Mertk*^*-/-*^ *vs* wild type
*Pcna-ps2*	20.16	18.10	6.69
*Gatm*	16.49	16.82	3.96
*Exd1*	4.60	4.21	4.09
*Gm18562*	3.84	4.47	3.53
*Steap3*	3.28	3.48	2.27
*Eif3j2*	2.91	2.85	2.49
*Gm47469*	2.19	2.21	3.68
*Zfp69*	2.54	3.23	1.98
*Tcp11l1*	2.12	2.94	2.10
*Atp10d*	2.47	2.50	2.00
*Csde1*	2.25	2.24	2.17
*Gm3650*	2.06	2.08	2.39
*Nras*	2.16	2.14	2.02
*Gpr137b*	1.98	2.06	2.22
*Sike1*	2.00	2.03	1.96
*Dynlt1b*	2.00	2.01	1.79
*Tmem181b-ps*	1.83	1.72	1.88
*Sptbn5*	3.05	3.01	-1.65
*Akr1e1*	-1.53	-1.84	-1.64
*D2hgdh*	-1.61	-1.63	-1.81
*H2bc8*	-1.62	-1.72	-2.01
*Zfp967*	-1.56	-1.63	-2.17
*Zfp968*	-1.68	-1.59	-2.10
*Ide*	-1.55	-1.69	-2.16
*Zfp932*	-1.62	-1.86	-1.93
*Zfp966*	-1.65	-1.67	-2.17
*Zfp969*	-1.77	-1.68	-2.06
*Eif3j1*	-2.04	-2.08	-1.76
*Idua*	-2.22	-2.41	-1.56
*Fgfbp3*	-2.09	-2.21	-1.97
*H2bc4*	-2.26	-2.29	-1.98
*Bub1b*	-2.61	-2.62	-1.63
*Mertk*	-2.55	-2.35	-1.98
*Stbd1*	-3.43	-4.32	-2.20
*Gpr137b-ps*	-3.34	-3.17	-4.14
*Bfsp1*	-3.71	-3.96	-3.95
*Jmjd7*	-16.85	-18.70	-8.34

The gene symbols (first column) and fold changes of DEGs identified in naïve, HRB-*Mertk*^*-/-*^ alveolar macrophages isolated from female (second column) or male (third column) mice and naïve lung tissue of *Mertk*^*-/-*^ male mice (fourth column). Genes are organized by average fold change among the three comparisons from most upregulated to downregulated.

*Comparison of pneumonic tissue between HRB-Mertk*^*-/-*^ *and wild type mice* Direct comparison of pneumonic lung tissue of HRB-*Mertk*^*-/-*^ and wild type mice identified 154 upregulated and 245 downregulated DEGs (S17 Tab in S2 File). Functional enrichment analysis of all DEGs in HRB-*Mertk*^*-/-*^ pneumonic lung tissue identified 15 GO terms and 32 Reactome pathways. GO terms enriched in HRB-*Mertk*^*-/-*^ tissue included positive regulation of defense response, leukocyte activation, and response to external stimuli. Reactome pathways identified describe regulation of the phototransduction cascade, transcriptional regulation, response to oxidative stress and lipid clearance (S18 Tab in S2 File).

**Fig 12:**
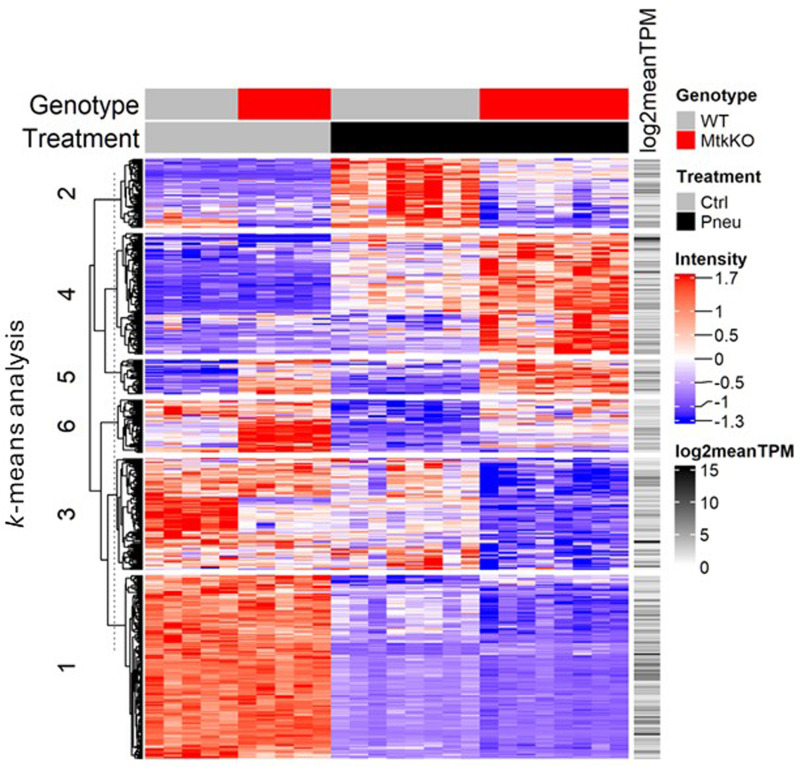
The left lung transcriptome is altered in both naïve and infected HRB-*Mertk*^*-/-*^ mice. Transcriptomic analysis was performed on naïve and pneumonic left lung tissues isolated from male HRB-*Mertk*^*-/-*^ and wild type mice (naïve: *n = *5 per genotype; pneumonic: *n = *8 per genotype, collected from two independent experiments). The number of inoculated bacteria was based on body weight (2.95 ± 0.52x10^7^ CFU/mL). As described in the methods, a differentially expressed gene (DEG) was defined as average expression >  0, FDR < 0.05, and fold change>  ± 1.5. (**A**) The heatmap depicts hierarchical clustering and *k-*means analysis using Euclidean distance metric of DEGs. The dendrogram (left) indicates clustering across transcripts (log2 normalized intensity) and each column represents an individual animal of the indicated genotype and treatment (top).

Hierarchical clustering and *k*-means analysis of DEGs identified in both naïve and pneumonic lung tissue revealed 6 gene clusters (Fig 12). Gene list over-representation using DEGs identified within each cluster found enriched GO processes in clusters 2 and 4 (S19 Tab in S2 File). Genes in cluster 2 were similar in naïve mice of either genotype and, increased only or to a much greater extent in wild type mice. Genes in this cluster are associated with oxidative stress and cellular metabolism. Genes in cluster 4 were also similar in naïve HRB-*Mertk*^*-/-*^ and wild type lung tissue but were upregulated to a greater extent in HRB-*Mertk*^*-/-*^ lungs during infection*.* GO terms associated with cluster 4 include regulation of pro-inflammatory pathways, response to bacterium, and cytokine production, including IFNγ and IL-6.

*Comparison of pneumonic to naïve lung tissue for each genotype* In HRB-*Mertk*^*-/-*^ mice, 7,009 genes were altered by *S. pneumoniae*, 3,196 upregulated and 3,813 downregulated (S20 Tab in S2 File). In wild type mice, 6,743 genes were altered by *S. pneumoniae*, 3,120 upregulated and 3,623 downregulated (S21 Tab in S2 File). Of the total DEGs identified, 5,991 were changed in both genotypes, 752 were changed uniquely in wild type mice, and 1,018 were changed in HRB-*Mertk*^*-/-*^ mice. Functional enrichment analysis identified 846 enriched GO terms in pneumonic HRB-*Mertk*^*-/-*^ mice (S22 Tab in S2 File) and 810 in wild type (S23 Tab in S2 File) post inoculation; 144 were unique to HRB-*Mertk*^*-/-*^ mice (S22 Tab in S2 File, rows 706-859) and 108 were unique to wild type mice (S23 Tab in S2 File, rows 706-823). Comparably, 206 Reactome pathways were identified in HRB-*Mertk*^*-/-*^ mice (S24 Tab in S2 File) and 208 in wild type (S25 Tab in S2 File); 33 were unique to HRB-*Mertk*^*-/-*^ mice (S24 Tab in S2 File, rows 177-219) and 35 to wild type (S25 Tab in S2 File, rows 177-221).

Functional enrichment pathways uniquely identified in either genotype were manually grouped into the same seven categories as for the transcriptomes of alveolar macrophages stimulated *ex vivo*. The pathways that were enriched in pneumonic HRB-*Mertk*^*-/-*^ lung tissue describe positive regulation of T cell proliferation and activation, NK cell mediated immunity, leukocyte migration, cellular motility and cell surface receptor signaling. Pathways enriched in pneumonic lung tissue of wild type mice were negative regulation of lymphocyte migration, regulation of T cell tolerance and of B cell apoptotic processes, and negative regulation of receptor signaling pathways via JAK-STAT.

DEGs induced by *S. pneumoniae* in either genotype were subsequently compared by identifying protein coding transcripts that show > 2-fold differences in expression. Among those DEGs identified in pneumonic lung tissue of both genotypes, 47 were > 2-fold different in HRB-*Mertk*^*-/-*^ mice and 20 in wild type mice. Of those DEGs uniquely identified in one genotype but not the other, 19 genes were expressed >  2-fold in HRB-*Mertk*^*-/-*^ mice and 18 in wild type mice. Individual genes and fold changes are listed in S26 Tab in S2 File.

To determine if there is any indication of enhanced phagocytosis in HRB-*Mertk*^*-/-*^ lungs, we searched the GO dataset of gene sets. The GSEA identified three GOs describing gene sets/pathways important in phagocytosis that are enriched in both genotypes, when compared to naïve mice of the same genotype (p <  0.02):

GO:0050764: regulation of phagocytosis
GO:0050766: positive regulation of phagocytosis
GO:0006909: phagocytosis

However, these GOs are not enriched in pneumonic lungs of HRB-*Mertk*^*-/-*^ compared to wild type lungs. In fact, of the 37 GO gene sets in the GO database that describe phagocytic processes, none are enriched in alveolar macrophages from HRB-*Mertk*^*-/-*^ compared to wild type mice. Furthermore, no differences in genes coding for pathogen recognition receptors are found between genotypes, and protein expression of CD93, the putative C1q receptor that is important in the membrane attack complex and in bacterial phagocytosis, is similar between genotypes (data not shown).

### Identification of DEGs within the 129P2 insertion present in HRB-*Mertk*^-/-^ mice

In an attempt to identify genes that may enhance bacterial clearance observed in HRB-*Mertk*^*-/-*^ mice, we identified the genes encoded in the 129P2 insertion on chromosome 2. There were 645 genes within the DNA insertion (S27A Tab in S2 File). This list was cross-referenced to DEGs present in naïve alveolar macrophages or naïve lung tissue of HRB-*Mertk*^*-/-*^ mice compared to wild type cells or lungs. Naïve alveolar macrophages from female or male HRB-*Mertk*^*-/-*^ had 22 or 19 DEGs identified within this region, respectively. In naïve lung tissue of male HRB-*Mertk*^*-/-*^, 20 DEGs were identified. These genes are summarized in S27B–D Tab in S2 File. There were 8 genes present in all three samples, and 8 other genes shared between alveolar macrophages. DEGs present in the DNA insert are listed and shown in a Venn diagram in S27E Tab in S2 File.

## Discussion

Our studies examined the inflammatory response to *S. pneumoniae*, a critically important pathogen, in the lungs of male and female wild type mice and in mice deficient in Mertk expression. Our data show that among lung macrophages, Mertk is expressed primarily by alveolar macrophages. HRB-*Mertk*^*-/-*^ mice demonstrate enhanced clearance of *S. pneumoniae*, as measured by the greater number of viable bacteria present in the lungs of wild type mice compared to HRB-*Mertk*^*-/-*^ mice 24 hours post-inoculation. This enhanced clearance was not present in CRISPR-*Mertk*^*-/-*^ mice, suggesting that this effect on clearance is not due solely to deficiency of Mertk, but is due to genomic differences between HRB-*Mertk*^*-/-*^ mice that retain segments of DNA of 129 mice and CRISPR-*Mertk*^*-/-*^ mice, in which the Mertk deletion was generated directly within the C57Bl/6J background.

The genotype and phenotype of the HRB-*Mertk*^*-/-*^ mice is complex. HRB-*Mertk*^*-/-*^ mice, the only commercially available Mertk null mouse, was studied in papers by Vollrath and Akalu [20,21]. Mertk is absent from these mice, but their genome contains an unclear number of DNA inserts from the 129P2 background [20,21]. Vollrath and colleagues noted the heterogeneity of the phenotype and identified a large DNA insert on chromosome 2 flanking *Mertk* [20]. Akalu and colleagues further described the widespread alternations in the genome of the HRB-*Mertk*^*-/-*^ mice and generated new *Mertk*^*-/-*^ knockouts using CRISPR/Cas9 technology [21]. Some of the phenotypes are similar between the HRB-*Mertk*^*-/-*^ and *Mertk*^*-/-*^ mice created using CRISPR/Cas9 technology [21]. Earp and colleagues created a Mertk^fl/fl^ mouse by CRISPR/Cas9 technology and reported that Mertk deletion in CD11c-expressing cells using a CD11c-Cre mouse resulted in the reversal of a dendritic cell dysfunctional/tolerizing phenotype [38].

As indicated in the Methods, Mertk^fl/fl^ mice were used to directly create the new CRISPR-*Mertk*^*-/-*^ mouse, and our studies described here show these mice did not have the enhanced bacterial clearance seen in HRB-*Mertk*^*-/-*^ mice. The current studies are beginning to unravel the mechanistic differences in the two mouse strains, as the phenotypes displayed appear to be both tissue and potentially functionally context specific. We have begun investigations of the 129 DNA insert on chromosome 2. Among the 645 genes we found in this insert, a total of 34 genes were differentially expressed by naïve male or female alveolar macrophages and lung tissue of HRB-*Mertk*^*-/-*^ mice compared to wild type mice. Our studies cannot yet address whether any of the DEGs account for the phenotype of the HRB-*Mertk*^*-/-*^ mice, or whether the phenotype is due to a single gene or a complex interaction between two or more genes.

The enhanced clearance observed in HRB-*Mertk*^*-/-*^ mice is not due to altered numbers of myeloid cells in the lung of naïve animals or at 24 hours post-inoculation. Significantly fewer neutrophils were present within the airspace of infected HRB-*Mertk*^*-/-*^ mice. Whether this difference is due to less neutrophil recruitment and/or more rapid resolution of the accumulated neutrophils is not determined. However, the most likely circumstance based on all our data is that HRB-*Mertk*^*-/-*^ mice destroy the bacteria more rapidly and thus induce less neutrophil recruitment. Assessment of inflammatory mediators in the BAL fluid identified significantly higher concentrations of IFNγ in HRB-*Mertk*^*-/-*^ mice of both sexes. However, neutralization of IFNγ did not prevent the enhanced bacterial clearance. Thus, IFNγ alone is not responsible for the more rapid loss of bacteria in the lungs of HRB-*Mertk*^*-/-*^ mice.

Our studies were powered to compare males and females for most parameters. Mice of the same age were studied; females weighed approximately 20% less than males. The inoculum of *S. pneumoniae* was adjusted for body weight. Thus, any differences between sexes is not due to a difference in the size of the stimulus. In naïve mice, there were more neutrophils and alveolar macrophages in the lung digests of males compared to females, as well as a trend toward more interstitial macrophages that did not reach significance. This difference is most likely due to the greater lung size in males compared to females. Interestingly, in WT mice with *S. pneumoniae* pneumonia, there were few differences between males and females in their response to *S. pneumoniae* at 24 hours post inoculation that can be attributed to an intrinsic difference in the immune response. In particular, there was no difference in clearance of *S. pneumoniae* between males and females. The pneumonic lung digests of males contained more neutrophils and inflammatory macrophages, also most likely due to their larger lungs. Male lungs had higher protein concentrations. This difference likely reflects use of the same lavage volume in both sexes, leading to greater dilution in the female samples that sometimes reached significance. The concentrations of cytokines often also followed this pattern, although female wild type mice with pneumonia did have increased concentrations of MCP-1, TNF-α and IL-12 (p40) compared to males of the same genotype. Thus, differences between the sexes appear due mostly to the larger lung size of male mice, rather than an intrinsic difference in their innate immune response.

Amongst myeloid cells, Mertk is expressed primarily on alveolar macrophages. It is also expressed on a small number of interstitial macrophages at a low intensity, and not detected on inflammatory macrophages, neutrophils or NK cells in the lung of naïve wild type animals (Fig 1, S2 Fig in S1 File). Mertk has an important function as an efferocytotic receptor on tissue resident macrophages [39]. In previous murine studies, Fujimori et al. also observed Mertk expression on airway macrophages obtained by lavage, but not on monocytes or monocyte-like macrophages in the lungs of naïve wild type mice [40]. Mohning et al. observed higher Mertk expression on resident alveolar macrophages versus recruited macrophages following LPS-induced lung injury [41]. Our data expand the description of Mertk expression on macrophage subpopulations in the lung using current classifications and focused our subsequent studies on the alveolar macrophage as an important cell type in the observed phenotype.

Alveolar macrophages act as a first line of defense against inhaled pathogens and particulate matter and are important regulators of the inflammatory response in the distal airways [42,43]. We find that in HRB-*Mertk*^*-/-*^ mice, alveolar macrophages undergo transcriptomic changes that are associated with GO terms describing leukocyte migration, inflammatory signaling, and the response to reactive oxygen species. In the naïve lung, surprisingly few transcriptomic changes are identified in HRB-*Mertk*^*-/-*^ mice. Of interest are the 37 DEGs found in both HRB-*Mertk*^*-/-*^ naïve alveolar macrophages and naïve lung tissues. The functions of these genes include metabolic processes (e.g., creatine biosynthetic pathway, glycogen metabolism, ATP binding/hydrolysis activity, intracellular transport), signal transduction (e.g., regulator of interferon response elements, fibroblast growth factor receptor signaling, mediators of ion transport, GTPase proteins/cofactors) and transcriptional regulation (e.g., zinc finger proteins, translation initiation factor subunits, histone subunits). Some of these functions may be attributed to Mertk deletion, but others are likely the result of the complex genomic alterations.

When alveolar macrophages were stimulated with *S. pneumoniae ex vivo*, transcriptomic changes in HRB-*Mertk*^*-/-*^ alveolar macrophages included increased expression of proinflammatory genes, such as Il1b, Tnf, Cxcl1, Cxcl2, and Cxcl3, compared to wild type cells. GO terms associated with the HRB-*Mertk*^*-/-*^ transcriptome included the positive regulation of adaptive immunity, cellular ion homeostasis, response to reactive oxygen species, cellular metabolism, and positive regulation of inflammatory signaling. Previous *in vitro* studies (first reported in the original description of the HRB-*Mertk*^*-/-*^ mice [22]) have shown that Mertk-signaling inhibits NFκB phosphorylation and DNA binding activity and the production of the inflammatory mediators TNF-α, IL-6 and IL-1β [22,44,45].

However, our studies provide no evidence that macrophages are actually the source of the enhanced clearance of *S. pneumoniae*. Phagocytosis and acidification of pHrodo-labeled *S. aureus* bioparticles *in vivo* by alveolar macrophages was similar between HRB-*Mertk*^*-/-*^ and wild type mice. Both the percentage of macrophages that phagocytosed and the median fluorescent intensity (MFI) of bioparticles within each macrophage did not differ between genotypes. *S. aureus* and *S. pneumoniae* are both Gram-positive cocci in the Staphylococcus genus and recognize similar pattern recognition receptors. Gene expression studies of alveolar macrophages from HRB-*Mertk*^*-/-*^ and wild type mice also offer no evidence that Mertk deficiency results in alterations in the machinery needed for phagocytosis. For example, gene set enrichment analysis identified three GOs describing gene sets/pathways important in phagocytosis that are enriched similarly in both genotypes, when compared to naïve mice of the same genotype (p <  0.02). Of the 37 GO gene sets identified in a search of the GO database that describe phagocytic processes, none are enriched in alveolar macrophages from HRB-*Mertk*^*-/-*^ compared to wild type mice. No differences in genes coding for pathogen recognition receptors or in protein expression of CD93 are found between genotypes. Furthermore, measurement of cytokines, chemokines and other mediators in the airspace environment sampled by bronchoalveolar lavage revealed increases in molecules such as IFNγ, IL12 and IL17 in the HRB-*Mertk*^*-/-*^ mice, which are not generated by alveolar macrophages at this time point. Interestingly, there is also no difference in the formation of NETs, another mechanism of killing bacteria that macrophages may indirectly regulate, between genotypes. Taken together, the data presented in this manuscript suggest that the observed enhancement of clearance of *S. pneumoniae* in HRB-*Mertk*^*-/-*^ mice is not mediated through changes in intrinsic macrophage function and probably not through an extrinsic effect, although extrinsic effects cannot be fully excluded. The totality of changes in the extracellular environment resulting from the 129 DNA insert and the deletion of Mertk seems the most likely mechanism underlying the enhancement in clearance.

To understand this environmental effect more clearly, we also evaluated the transcriptomic changes induced by *S. pneumoniae* in pneumonic lung tissue of HRB-*Mertk*^*-/-*^ and wild type mice. Similar to the observations made in stimulated HRB-*Mertk*^*-/-*^ alveolar macrophages, these mice showed changes in the expression of genes that regulate the innate immune response, leading to the enrichment of unique GO terms. Pathways unique to the HRB-*Mertk*^*-/-*^ mice describe response to cell surface receptor signaling, tissue morphogenesis and ion transport. Functional enrichment pathways unique to wild type lung tissue identified a subset describing the regulation of JAK-STAT receptor signaling pathways. These observations suggest that in wild type mice, Mertk may act to repress inflammatory signaling [22] and delay clearance; however, the genomic alterations in the HRB-*Mertk*^*-/-*^ mice likely also contribute to these changes through molecules other than Mertk.

In summary, alveolar macrophages are constantly exposed to stimuli, both noxious and inert, including foreign particulate matter, microbes, apoptotic or necrotic debris, allergens and DAMP. Encountering these stimuli would lead to activation of signaling pathways, and Mertk may contribute to negatively regulate this activation in homeostatic environments. Other alterations in the genome of HRB-*Mertk*^*-/-*^ mice likely contribute to the observed priming of alveolar macrophages for a response to external stimuli. Following inoculation with *S. pneumoniae,* HRB-*Mertk*^*-/-*^ alveolar macrophages may be more motile than wild type cells, thus encountering and phagocytosing bacteria more rapidly. Based on transcriptomic evidence from *ex vivo* cultures, HRB-*Mertk*^*-/-*^ alveolar macrophages may also produce more proinflammatory mediators compared to wild type macrophages. Mertk deficiency may contribute to these changes, but because the enhanced bacterial clearance is seen only in HRB-*Mertk*^*-/-*^ mice and not in CRISPR-*Mertk*^*-/-*^ mice, the altered genome of the HRB-*Mertk*^*-/-*^ mice must be mediating at least some of this phenotype. The complexities of the genome of the HRB-*Mertk*^*-/-*^ mice make the mechanism of the enhanced clearance difficult to discern at this time. However, means of enhancing bacterial clearance are critically important as a therapeutic approach, and important targets may be identified as these HRB-*Mertk*^*-/-*^ mice become better understood.

## Supporting information

S1 File S1 Table: Antibodies used in flow cytometry studies. S2 Table: Original body weights, inoculum, and weight loss of animals infected with *S. pneumoniae.* The sample size (*n*), original body weights, number of *S. pneumonia* inoculated, body weight 24 hours post inoculation (24h PI) and percent original body weight of (A) animals evaluated in 24 hours post inoculation or (B) studies utilizing IFNγ neutralization, also studied 24 hours post inoculation (Fig 6). Statistically significant differences among each group were determined by one-way ANOVA followed by Bonferroni’s multiple comparisons test. The adjusted p values for selected comparisons and each metric are shown. Data represent mean ± SEM. **S1 Fig: Representative gating strategy utilized for flow cytometric analysis of left lung homogenates. S2 Fig: Characterization of immune cells present in the left lung of naïve HRB-*Mertk***^***-/-***^ **and wild type mice.** The (A) number of natural killer cells present in left lung homogenates of naïve HRB-*Mertk*^*-/-*^ and wild type mice were determined by flow cytometry. The (B) number of HRB-*Mertk*^+^ natural killer cells and (C) percentage of HRB-*Mertk*^+^ natural killer cells were determined. The (D) number of HRB-*Mertk*^+^ neutrophils and (E) percentage of HRB-*Mertk*^+^ neutrophils were derived from the total number of neutrophils (Fig 2C). Data are presented as mean ± SEM. Statistically significant differences evaluated by one-way ANOVA followed by Tukey’s multiple comparison test. **S3 Fig: Characteristics of naïve alveolar macrophages utilized for RNA sequencing studies.** The (**A**) total cell count and (**B**) differential cell analysis was determined following bilateral whole lung lavage. The total number of cells present in the BAL was similar between wild type and HRB-*Mertk*^*-/-*^ mice of both sexes. The majority of cells isolated in the BAL of wild type and HRB-*Mertk*^*-/-*^ mice were airway macrophages with very few lymphocytes, polymorphonuclear leukocytes (PMN), or other cell types. Data were collected from two independent experiments and are expressed as mean ± SEM.
(DOCX)

S2 File S1 Tab: Differentially expressed genes identified in naïve, female vs male alveolar macrophages. The table shows those DEGs identified in female *vs* male **(A)** wild type or **(B)** HRB-*Mertk*^*-/-*^ alveolar macrophage transcriptomes. No downregulated genes were identified. Asterisks indicate those genes identified in both genotypes. **S2 Tab: Differentially expressed genes identified in female, HRB-*Mertk***^***-/-***^ **vs wild type alveolar macrophages.** Comparison of naïve, HRB-*Mertk*^-/-^ vs wild type alveolar macrophages isolated from female mice. The table list those DEGs that changed>  ± 1.5-fold and are organized by fold change (Column J). **S3 Tab: Differentially expressed genes identified in male, HRB-*Mertk***^***-/-***^ **vs wild type alveolar macrophages.** Comparison of naïve, HRB-*Mertk*^-/-^ vs wild type alveolar macrophages isolated from male mice. The table list those DEGs that changed>  ± 1.5-fold and are organized by fold change (Column J). **S4 Tab: Gene ontology terms identified by functional enrichment analysis of HRB-*Mertk***^***-/-***^ **vs wild type alveolar macrophages.** Gene set enrichment analysis was performed using all expressed genes ranked by logFC in HRB-*Mertk*^-/-^ vs wild type alveolar macrophages. The table list those gene ontology terms enriched in HRB-*Mertk*^*-/-*^ *vs* wild type alveolar macrophage transcriptomes (adjPval <  0.05). Pathways are organized by normalized enrichment score, NES. **S5 Tab: Gene ontology terms identified by gene list over-representation analysis of DEGs in each *k*-means cluster comparing HRB-*Mertk***^***-/-***^ ***vs* wild type alveolar macrophages.** *k*-means clustering analysis was performed on DEGs that changed>  ± 1.5-fold in HRB-*Mertk*^*-/-*^ alveolar macrophages. The analysis resulted in 4 clusters. Gene list over-representation analysis using DEGs was performed within each cluster. The table list the statistically significant GO terms enriched in (**A**) cluster 1 (rows 3-21) and (**B**) cluster 4 (rows 24-43). No GO terms were identified in clusters 2 or 3. GO pathways are organized by adjusted *p* value (padj). The number of DEGs (overlap) identified in HRB-*Mertk*^*-/-*^ alveolar macrophages and their gene symbols (overlap genes) associated with each GO pathway are listed. (**C**) The DEGs used to perform *k-*means analysis and the clusters they were associated with are listed in column A. **S6 Tab: Differentially expressed genes identified in unstimulated, HRB-*Mertk***^***-/-***^ ***vs* wild type alveolar macrophages *ex vivo***. Comparison of unstimulated, *ex vivo* alveolar macrophages isolated from HRB-*Mertk*^*-/-*^ or wild type mice. The table list those DEGs that changed>  ± 1.5-fold and are organized by fold change. **S7 Tab: Differentially expressed genes identified in *S. pneumoniae* stimulated, HRB-*Mertk***^***-/-***^ ***vs* wild type alveolar macrophages *ex vivo***. Comparison of S. pneumoniae stimulated, *ex vivo* alveolar macrophages isolated from HRB-*Mertk*^*-/-*^ or wild type mice. The table list those DEGs that changed>  ± 1.5-fold and are organized by fold change. **S8 Tab: Gene ontology terms identified by functional enrichment analysis of *S. pneumoniae* stimulated, HRB-*Mertk***^***-/-***^ **vs wild type alveolar macrophages *ex vivo*.** Gene set enrichment analysis was performed using all expressed genes ranked by logFC in S. pneumoniae stimulated HRB-*Mertk*^*-/-*^ *vs* wild type *ex vivo* alveolar macrophages. The table list those gene ontology terms enriched in stimulated *Mertk*^*-/-*^ *vs* wild type alveolar macrophage transcriptomes (adjPval <  0.05). Pathways are organized by normalized enrichment score, NES. **S9 Tab: Differentially expressed genes identified in HRB-*Mertk***^***-/-***^**, *S. pneumoniae* stimulated *vs* unstimulated alveolar macrophages *ex vivo***. Comparison of *S. pneumoniae* stimulated vs unstimulated HRB-*Mertk*^*-/-*^ alveolar macrophages. The table list those DEGs that changed>  ± 1.5-fold and are organized by fold change. **S10 Tab: Differentially expressed genes identified in wild type, *S. pneumoniae* stimulated *vs* unstimulated alveolar macrophages *ex vivo*.** Comparison of *S. pneumoniae stimulated vs* unstimulated wild type alveolar macrophages. The table list those DEGs that changed>  ± 1.5-fold and are organized by fold change. **S11 Tab: Gene ontology terms identified by functional enrichment analysis of HRB-*Mertk***^***-/-***^**, *S. pneumoniae* stimulated *vs* unstimulated alveolar macrophages *ex vivo***. Gene set enrichment analysis was performed using all expressed genes ranked by logFC in *S. pneumonia* stimulated HRB-*Mertk*^*-/-*^ alveolar macrophages compared to unstimulated cells. The table list those gene ontology terms enriched in stimulated HRB-*Mertk*^*-/-*^ alveolar macrophage transcriptomes (adjPval <  0.05). Pathways similarly identified in stimulated *vs* unstimulated, wild type alveolar macrophages (S12 Tab) are highlighted in light yellow and are listed at the top of the table organized by normalized enrichment score (NES, rows 3-132). Pathways uniquely identified in *S. pneumoniae* stimulated HRB-*Mertk*^*-/-*^ alveolar macrophages are listed in rows 145-529. Those pathways were manually grouped into functional categories and are organized first by category and second by normalized enrichment score (NES). The key provided (rows 134-142) describes those functional categories. **S12 Tab: Gene ontology terms identified by functional enrichment analysis of wild type, *S. pneumoniae* stimulated vs unstimulated alveolar macrophages *ex vivo*.** Gene set enrichment analysis was performed using all expressed genes ranked by logFC in *S. pneumonia* stimulated wild type alveolar macrophages compared to unstimulated cells. The table list those gene ontology terms enriched in stimulated wild type alveolar macrophage transcriptomes (adjPval <  0.05). Pathways similarly identified in stimulated *vs* unstimulated, HRB-*Mertk*^*-/-*^ alveolar macrophages (S11 Tab in S2 File) are highlighted in light yellow and are listed at the top of the table organized by normalized enrichment score (NES, rows 3-132). Pathways uniquely identified in *S. pneumoniae* stimulated wild type alveolar macrophages are listed in rows 144-189. Those pathways were manually grouped into functional categories and are organized first by category and second by normalized enrichment score (NES). The key provided (rows 134-142) describes those functional categories. **S13 Tab: Reactome pathways identified by functional enrichment analysis of HRB-*Mertk***^***-/-***^**, *S. pneumoniae* stimulated *vs* unstimulated alveolar macrophages *ex vivo***. Gene set enrichment analysis was performed using all expressed genes ranked by logFC in *S. pneumonia* stimulated HRB-*Mertk*^*-/-*^ alveolar macrophages compared to unstimulated cells. The table list those Reactome pathways enriched in stimulated HRB-*Mertk*^*-/-*^ alveolar macrophage transcriptomes (adjPval <  0.05). Pathways similarly identified in stimulated *vs* unstimulated, wild type alveolar macrophages (Supplemental Table 14) are highlighted in light yellow and are listed at the top of the table organized by normalized enrichment score (NES, rows 3-43). Pathways uniquely identified in *S. pneumoniae* stimulated HRB-*Mertk*^*-/-*^ alveolar macrophages are listed in rows 55-94. Those pathways were manually grouped into functional categories and are organized first by category and second by normalized enrichment score (NES). The key provided (rows 45-53) describes those functional categories. **S14 Tab: Reactome pathway identified by functional enrichment analysis of wild type, *S. pneumoniae* stimulated *vs* unstimulated alveolar macrophages *ex vivo***. Gene set enrichment analysis was performed using all expressed genes ranked by logFC in *S. pneumonia* stimulated wild type alveolar macrophages compared to unstimulated cells. The table list those Reactome pathways enriched in stimulated wild type alveolar macrophage transcriptomes (adjPval <  0.05). Pathways similarly identified in stimulated *vs* unstimulated, HRB-*Mertk*^*-/-*^ alveolar macrophages (S13 Tab in S2 File) are highlighted in light yellow and are listed at the top of the table organized by normalized enrichment score (NES, rows 3-43). Pathways uniquely identified in *S. pneumoniae* stimulated wild type alveolar macrophages are listed in rows 55-79. Those pathways were manually grouped into functional categories and are organized first by category and second by normalized enrichment score (NES). The key provided (rows 45-53) describes those functional categories. **S15 Tab: Comparison of gene expression changes induced by *S. pneumoniae* in alveolar macrophages *ex vivo***. DEGs identified in S. pneumoniae stimulated vs unstimulated alveolar macrophages of either HRB-*Mertk*^*-/-*^ (S9 Tab in S2 File) or wild type (S10 Tab in S2 File) mice were compared by finding the ratio of observed fold changes. The table list (**A**) DEGs originally identified in both genotypes (rows 4-78), (**B**) DEGs only identified in wild type mice (rows 84-272) or (**C**) DEGs only identified in HRB-*Mertk*^*-/-*^ mice (rows 280-358) with a difference in expression >  2-fold (indicated by bold font). **S16 Tab: Differentially expressed genes identified in naïve lung tissue of HRB-*Mertk***^***-/-***^ ***vs* wild type mice.** Comparison of HRB-*Mertk*^*-/-*^ *vs* wild type naïve left lung tissue. The table list those DEGs that changed>  ± 1.5-fold and are organized by fold change. Asterisks indicate those DEGs similarly identified in *Mertk*^-/-^ alveolar macrophages. **S17 Tab: Differentially expressed genes identified in pneumonic lung tissue of HRB-*Mertk***^**-/-**^ **vs wild type mice.** Comparison of HRB-*Mertk*^*-/-*^ *vs* wild type pneumonic left lung tissue. The table list those DEGs that changed>  ± 1.5-fold and are organized by fold change. **S18 Tab: Gene ontology terms and Reactome pathways identified by functional enrichment analysis of pneumonic, HRB-*Mertk***^**-/-**^ **vs wild type left lung tissue.** Gene set enrichment analysis was performed using all expressed genes ranked by logFC in pneumonic, HRB-*Mertk*^*-/-*^ *vs* wild type left lung tissue. The table list (**A**) gene ontology terms and (**B**) Reactome pathways enriched in HRB-*Mertk*^*-/-*^ tissue (adjPval <  0.05) and are organized by normalized enrichment scores (NES). **S19 Tab: Gene ontology terms identified by gene list over-representation analysis of DEGs in each *k*-means cluster comparing HRB-*Mertk***^***-/-***^ ***vs* wild type left lung tissue.** *k*-means clustering analysis was performed on DEGs that changed>  ± 1.5-fold in HRB-*Mertk*^*-/-*^ lung tissue. The analysis resulted in 6 clusters. Gene list over-representation analysis using DEGs was performed within each cluster. The table lists the statistically significant gene ontology terms enriched in (**A**) cluster 2 (rows 7-9) and (**B**) cluster 4 (rows 15-51). No GO terms were identified in clusters 1, 3, 5 or 6. GO pathways are organized by adjusted *p* value (padj). The number of DEGs (overlap) and their gene symbols (overlap genes) which were associated with each GO pathway are listed. (**C**) The DEGs used to perform *k-*means analysis and the clusters which they are associated with are listed (Column A, rows 56-487). **S20 Tab: Differentially expressed genes identified in HRB-*Mertk***^***-/-***^**, pneumonic *vs* naïve left lung tissue.** Comparison of pneumonic *vs* naïve, left lung tissue isolated from HRB-*Mertk*^*-/-*^ mice. The table list those DEGs that changed>  ± 1.5-fold and are organized by fold change. **S21 Tab: Differentially expressed genes identified in wild type, pneumonic *vs* naïve left lung tissue.** Comparison of pneumonic *vs* naïve, left lung tissue isolated from wild type mice. The table list those DEGs that changed>  ± 1.5-fold and are organized by fold change. **S22 Tab: Gene ontology terms identified by functional enrichment analysis of HRB-*Mertk***^***-/-***^**, pneumonic *vs* naïve left lung tissue.** Gene set enrichment analysis was performed using all expressed genes ranked by logFC in pneumonic *vs* naïve HRB-*Mertk*^*-/-*^ lung tissue. The table list those gene ontology terms enriched in pneumonic HRB-*Mertk*^*-/-*^ lung tissue (adjPval <  0.05). Pathways similarly identified in pneumonic *vs* naïve, wild type lung tissue (S23 Tab in S2 File) are highlighted in light yellow and are listed at the top of the table organized by normalized enrichment score (NES, rows 3-704). Pathways uniquely identified in pneumonic HRB-*Mertk*^*-/-*^ lung tissue are listed in rows 716-859. Those pathways were manually grouped into functional categories and are organized first by category and second by normalized enrichment score (NES). The key provided (rows 706-714) describes those functional categories. **S23 Tab: Gene ontology terms identified by functional enrichment analysis of wild type, pneumonic *vs* naïve left lung tissue.** Gene set enrichment analysis was performed using all expressed genes ranked by logFC in pneumonic *vs* naïve wild type lung tissue. The table list those gene ontology terms enriched in pneumonic wild type lung tissue (adjPval <  0.05). Pathways similarly identified in pneumonic *vs* naïve, wild type lung tissue (S22 Tab in S2 File) are highlighted in light yellow and are listed at the top of the table organized by normalized enrichment score (NES, rows 3-704). Pathways uniquely identified in pneumonic wild type lung tissue are listed in rows 716-823. Those pathways were manually grouped into functional categories and are organized first by category and second by normalized enrichment score (NES). The key provided (rows 706-714) describes those functional categories. **S24 Tab: Reactome pathways identified by functional enrichment analysis of HRB-*Mertk***^***-/-***^**, pneumonic *vs* naïve left lung tissue.** Gene set enrichment analysis was performed using all expressed genes ranked by logFC in pneumonic *vs* naïve HRB-*Mertk*^*-/-*^ lung tissue. The table list those Reactome pathways enriched in pneumonic HRB-*Mertk*^*-/-*^ lung tissue (adjPval <  0.05). Pathways similarly identified in pneumonic *vs* naïve, wild type lung tissue (Supplemental Table 25) are highlighted in light yellow and are listed at the top of the table organized by normalized enrichment score (NES, rows 3-175). Pathways uniquely identified in pneumonic HRB-*Mertk*^*-/-*^ lung tissue are listed in rows 187-219. Those pathways were manually grouped into functional categories and are organized first by category and second by normalized enrichment score (NES). The key provided (rows 177-185) describes those functional categories. **S25 Tab: Reactome pathways identified by functional enrichment analysis of wild type, pneumonic *vs* naïve left lung tissue.** Gene set enrichment analysis was performed using all expressed genes ranked by logFC in pneumonic *vs* naïve wild type lung tissue. The table list those Reactome pathways enriched in pneumonic wild type lung tissue (adjPval <  0.05). Pathways similarly identified in pneumonic vs naïve, HRB-*Mertk*^*-/-*^ lung tissue (Supplemental Table 24) are highlighted in light yellow and are listed at the top of the table organized by normalized enrichment score (NES, rows 3-175). Pathways uniquely identified in pneumonic wild type lung tissue are listed in rows 187-221. Those pathways were manually grouped into functional categories and are organized first by category and second by normalized enrichment score (NES). The key provided (rows 177-185) describes those functional categories. **S26 Tab: Comparison of gene expression changes induced by *S. pneumoniae* in left lung tissue.** DEGs identified in pneumonic *vs* naïve lung tissue of either HRB-*Mertk*^*-/-*^ (S21 Tab in S2 File) or wild type (S20 Tab in S2 File) mice were compared by finding the ratio of observed fold changes. The table list (**A**) DEGs identified in both genotypes (rows 4-71), (**B**) DEGs only identified in wild type mice (rows 74-91) or (**C**) DEGs only identified in HRB-*Mertk*^*-/-*^ mice (rows 98-116) with a difference in expression >  2-fold (indicated by bold font). **S27 Tab: Genes known to be located within the 129P2 insertion region on chromosome 2 and DEGs identified in naïve alveolar macrophages or lung tissue of HRB-*Mertk***^***-/-***^ **vs. wild type mice. (A)** Genes located within the 129P2 DNA insert of chromosome 2. Listed in order of chromStart. DEGs identified in (**B**) naïve female alveolar macrophages, (**C**) male alveolar macrophages or (**D**) lung tissue of HRB-*Mertk*^*-/-*^ vs. wild type mice. (**E**) Venn diagram comparing DEGs identified in alveolar macrophages and lung tissue.
(XLSX)

## References

[1] Dela Cruz CS, Wunderink RG, Christiani DC, Cormier SA, Crothers K, Doerschuk CM, et al. Future Research Directions in Pneumonia. NHLBI Working Group Report. Am J Respir Crit Care Med. 2018;198(2):256–63. doi: 10.1164/rccm.201801-0139WS 29546996 PMC6058989

[2] Quinton LJ, Walkey AJ, Mizgerd JP. Integrative Physiology of Pneumonia. Physiol Rev. 2018;98(3):1417–64. doi: 10.1152/physrev.00032.2017 29767563 PMC6088146

[3] Koulenti D, Zhang Y, Fragkou PC. Nosocomial pneumonia diagnosis revisited. Curr Opin Crit Care. 2020;26(5):442–9. doi: 10.1097/MCC.0000000000000756 32739969

[4] Metlay JP, Waterer GW. Update in adult community-acquired pneumonia: key points from the new American Thoracic Society/Infectious Diseases Society of America 2019 guideline. Curr Opin Pulm Med. 2020;26(3):203–7. doi: 10.1097/MCP.0000000000000671 32084039

[5] Kumar V. Pulmonary Innate Immune Response Determines the Outcome of Inflammation During Pneumonia and Sepsis-Associated Acute Lung Injury. Front Immunol. 2020;11:1722. doi: 10.3389/fimmu.2020.01722 32849610 PMC7417316

[6] Quinton LJ, Mizgerd JP. Dynamics of lung defense in pneumonia: resistance, resilience, and remodeling. Annu Rev Physiol. 2015;77:407–30. doi: 10.1146/annurev-physiol-021014-071937 25148693 PMC4366440

[7] Mizgerd JP. Respiratory infection and the impact of pulmonary immunity on lung health and disease. Am J Respir Crit Care Med. 2012;186(9):824–9. doi: 10.1164/rccm.201206-1063PP 22798317 PMC3530220

[8] Korkmaz FT, Traber KE. Innate immune responses in pneumonia. Pneumonia (Nathan). 2023;15(1):4. doi: 10.1186/s41479-023-00106-8 36829255 PMC9957695

[9] Li D, Wu M. Pattern recognition receptors in health and diseases. Signal Transduct Target Ther. 2021;6(1):291. doi: 10.1038/s41392-021-00687-0 34344870 PMC8333067

[10] Koppe U, Suttorp N, Opitz B. Recognition of Streptococcus pneumoniae by the innate immune system. Cell Microbiol. 2012;14(4):460–6. doi: 10.1111/j.1462-5822.2011.01746.x 22212419

[11] Hagan RS, Gomez JC, Torres-Castillo J, Martin JR, Doerschuk CM. TBK1 Is Required for Host Defense Functions Distinct from Type I IFN Expression and Myeloid Cell Recruitment in Murine Streptococcus pneumoniae Pneumonia. Am J Respir Cell Mol Biol. 2022;66(6):671–81. doi: 10.1165/rcmb.2020-0311OC 35358404 PMC9163639

[12] Gil E, Noursadeghi M, Brown JS. Streptococcus pneumoniae interactions with the complement system. Front Cell Infect Microbiol. 2022;12:929483. doi: 10.3389/fcimb.2022.929483 35967850 PMC9366601

[13] Graham DK, DeRyckere D, Davies KD, Earp HS. The TAM family: phosphatidylserine sensing receptor tyrosine kinases gone awry in cancer. Nat Rev Cancer. 2014;14(12):769–85. doi: 10.1038/nrc3847 25568918

[14] Lemke G. Biology of the TAM receptors. Cold Spring Harb Perspect Biol. 2013;5(11):a009076. doi: 10.1101/cshperspect.a009076 24186067 PMC3809585

[15] Rothlin CV, Carrera-Silva EA, Bosurgi L, Ghosh S. TAM receptor signaling in immune homeostasis. Annu Rev Immunol. 2015;33:355–91. doi: 10.1146/annurev-immunol-032414-112103 25594431 PMC4491918

[16] Lew ED, Oh J, Burrola PG, Lax I, Zagórska A, Través PG, et al. Differential TAM receptor-ligand-phospholipid interactions delimit differential TAM bioactivities. Elife. 2014;3:e03385. doi: 10.7554/eLife.03385 25265470 PMC4206827

[17] Dransfield I, Zagórska A, Lew ED, Michail K, Lemke G. Mer receptor tyrosine kinase mediates both tethering and phagocytosis of apoptotic cells. Cell Death Dis. 2015;6(2):e1646. doi: 10.1038/cddis.2015.18 25695599 PMC4669813

[18] Ravichandran KS. Find-me and eat-me signals in apoptotic cell clearance: progress and conundrums. J Exp Med. 2010;207(9):1807–17. doi: 10.1084/jem.20101157 20805564 PMC2931173

[19] Scott RS, McMahon EJ, Pop SM, Reap EA, Caricchio R, Cohen PL, et al. Phagocytosis and clearance of apoptotic cells is mediated by MER. Nature. 2001;411(6834):207–11. doi: 10.1038/35075603 11346799

[20] Vollrath D, Yasumura D, Benchorin G, Matthes MT, Feng W, Nguyen NM, et al. Tyro3 Modulates Mertk-Associated Retinal Degeneration. PLoS Genet. 2015;11(12):e1005723. doi: 10.1371/journal.pgen.1005723 26656104 PMC4687644

[21] Akalu YT, Mercau ME, Ansems M, Hughes LD, Nevin J, Alberto EJ, et al. Tissue-specific modifier alleles determine Mertk loss-of-function traits. Elife. 2022;11:e80530. doi: 10.7554/eLife.80530 35969037 PMC9433089

[22] Camenisch TD, Koller BH, Earp HS, Matsushima GK. A novel receptor tyrosine kinase, Mer, inhibits TNF-alpha production and lipopolysaccharide-induced endotoxic shock. J Immunol. 1999;162(6):3498–503. doi: 10.4049/jimmunol.162.6.3498 10092806

[23] Sharma-Chawla N, Stegemann-Koniszewski S, Christen H, Boehme JD, Kershaw O, Schreiber J, et al. In vivo Neutralization of Pro-inflammatory Cytokines During Secondary Streptococcus pneumoniae Infection Post Influenza A Virus Infection. Front Immunol. 2019;10:1864. doi: 10.3389/fimmu.2019.01864 31474978 PMC6702285

[24] Nakamatsu M, Yamamoto N, Hatta M, Nakasone C, Kinjo T, Miyagi K, et al. Role of interferon-gamma in Valpha14+ natural killer T cell-mediated host defense against Streptococcus pneumoniae infection in murine lungs. Microbes Infect. 2007;9(3):364–74. doi: 10.1016/j.micinf.2006.12.003 17314060

[25] Dobin A, Davis CA, Schlesinger F, Drenkow J, Zaleski C, Jha S, et al. STAR: ultrafast universal RNA-seq aligner. Bioinformatics. 2013;29(1):15–21. doi: 10.1093/bioinformatics/bts635 23104886 PMC3530905

[26] Pertea M, Pertea GM, Antonescu CM, Chang T-C, Mendell JT, Salzberg SL. StringTie enables improved reconstruction of a transcriptome from RNA-seq reads. Nat Biotechnol. 2015;33(3):290–5. doi: 10.1038/nbt.3122 25690850 PMC4643835

[27] Law CW, Chen Y, Shi W, Smyth GK. voom: Precision weights unlock linear model analysis tools for RNA-seq read counts. Genome Biol. 2014;15(2):R29. doi: 10.1186/gb-2014-15-2-r29 24485249 PMC4053721

[28] Ritchie ME, Phipson B, Wu D, Hu Y, Law CW, Shi W, et al. limma powers differential expression analyses for RNA-sequencing and microarray studies. Nucleic Acids Res. 2015;43(7):e47. doi: 10.1093/nar/gkv007 25605792 PMC4402510

[29] Subramanian A, Tamayo P, Mootha VK, Mukherjee S, Ebert BL, Gillette MA, et al. Gene set enrichment analysis: a knowledge-based approach for interpreting genome-wide expression profiles. Proc Natl Acad Sci USA. 2005;102(43):15545–50. doi: 10.1073/pnas.0506580102 16199517 PMC1239896

[30] Korotkevich G, Sukhov V, Budin N, Shpak B, Artyomov MN, Sergushichev A. Fast gene set enrichment analysis 2016.

[31] Ashburner M, Ball CA, Blake JA, Botstein D, Butler H, Cherry JM, et al. Gene ontology: tool for the unification of biology. The Gene Ontology Consortium. Nat Genet. 2000;25(1):25–9. doi: 10.1038/75556 10802651 PMC3037419

[32] Wu G, Haw R. Functional Interaction Network Construction and Analysis for Disease Discovery. Methods Mol Biol. 2017;1558:235–53. doi: 10.1007/978-1-4939-6783-4_11 28150241

[33] Heberle H, Meirelles GV, da Silva FR, Telles GP, Minghim R. InteractiVenn: a web-based tool for the analysis of sets through Venn diagrams. BMC Bioinformatics. 2015;16(1):169. doi: 10.1186/s12859-015-0611-3 25994840 PMC4455604

[34] Gu Z, Eils R, Schlesner M. Complex heatmaps reveal patterns and correlations in multidimensional genomic data. Bioinformatics. 2016;32(18):2847–9. doi: 10.1093/bioinformatics/btw313 27207943

[35] Kent WJ. BLAT--the BLAST-like alignment tool. Genome Res. 2002;12(4):656–64. doi: 10.1101/gr.229202 11932250 PMC187518

[36] Karolchik D, Hinrichs AS, Furey TS, Roskin KM, Sugnet CW, Haussler D, et al. The UCSC Table Browser data retrieval tool. Nucleic Acids Res. 2004;32(Database issue):D493-6. doi: 10.1093/nar/gkh103 14681465 PMC308837

[37] McPeek MK, Gomez JC, Martin JR, Iannone MA, Dang H, Doerschuk CM. Leukocyte kinetics and bacterial clearance during Streptococcus pneumoniae pneumonia and contributions of ICAM-1. Am J Physiol Lung Cell Mol Physiol. 2025;328(1):L41–59. doi: 10.1152/ajplung.00039.2024 39437756 PMC11905799

[38] Zewdie EY, Edwards GM, Hunter DM, Earp HS, Holtzhausen A. MerTK Induces Dysfunctional Dendritic Cells by Metabolic Reprogramming. Cancer Immunol Res. 2024;12(9):1268–85. doi: 10.1158/2326-6066.CIR-23-0666 38976507 PMC11371516

[39] Lemke G. How macrophages deal with death. Nat Rev Immunol. 2019;19(9):539–49. doi: 10.1038/s41577-019-0167-y 31019284 PMC6733267

[40] Fujimori T, Grabiec AM, Kaur M, Bell TJ, Fujino N, Cook PC, et al. The Axl receptor tyrosine kinase is a discriminator of macrophage function in the inflamed lung. Mucosal Immunol. 2015;8(5):1021–30. doi: 10.1038/mi.2014.129 25603826 PMC4430298

[41] Mohning MP, Thomas SM, Barthel L, Mould KJ, McCubbrey AL, Frasch SC, et al. Phagocytosis of microparticles by alveolar macrophages during acute lung injury requires MerTK. Am J Physiol Lung Cell Mol Physiol. 2018;314(1):L69–82. doi: 10.1152/ajplung.00058.2017 28935638 PMC6335009

[42] Neupane AS, Willson M, Chojnacki AK, Vargas E Silva Castanheira F, Morehouse C, Carestia A, et al. Patrolling Alveolar Macrophages Conceal Bacteria from the Immune System to Maintain Homeostasis. Cell. 2020;183(1):110-125.e11. doi: 10.1016/j.cell.2020.08.020 32888431

[43] Hussell T, Bell TJ. Alveolar macrophages: plasticity in a tissue-specific context. Nat Rev Immunol. 2014;14(2):81–93. doi: 10.1038/nri3600 24445666

[44] Zhang B, Fang L, Wu H-M, Ding P-S, Xu K, Liu R-Y. Mer receptor tyrosine kinase negatively regulates lipoteichoic acid-induced inflammatory response via PI3K/Akt and SOCS3. Mol Immunol. 2016;76:98–107. doi: 10.1016/j.molimm.2016.06.016 27419619

[45] Alciato F, Sainaghi PP, Sola D, Castello L, Avanzi GC. TNF-alpha, IL-6, and IL-1 expression is inhibited by GAS6 in monocytes/macrophages. J Leukoc Biol. 2010;87(5):869–75. doi: 10.1189/jlb.0909610 20103767

