# Supplementary material for: Host responses to S. pneumoniae in wild type and Mertk mutant mice
Source: PLoS One. 2025 Apr 16;20(4):e0320660. doi: 10.1371/journal.pone.0320660 (PMC12002534; doi:10.1371/journal.pone.0320660)
Supplement: S1 File — The sample size (n), original body weights, number of S. pneumonia inoculated, body weight 24 hours post inoculation (24h PI) and percent original body weight of (A) animals evaluated in 24 hours post inoculation or (B) studies utilizing IFNγ neutralization, also studied 24 hours post inoculation (Fig 6). Statistically significant differences among each group were determined by one-way ANOVA followed by Bonferroni’s multiple comparisons test. The adjusted p values for selected comparisons and each metric are shown. Data represent mean ± SEM. S1 Fig: Representative gating strategy utilized for flow cytometric analysis of left lung homogenates. S2 Fig: Characterization of immune cells present in the left lung of naïve HRB-Mertk-/- and wild type mice. The (A) number of natural killer cells present in left lung homogenates of naïve HRB-Mertk-/- and wild type mice were determined by flow cytometry. The (B) number of HRB-Mertk+ natural killer cells and (C) percentage of HRB-Mertk+ natural killer cells were determined. The (D) number of HRB-Mertk+ neutrophils and (E) percentage of HRB-Mertk+ neutrophils were derived from the total number of neutrophils (Fig 2C). Data are presented as mean ± SEM. Statistically significant differences evaluated by one-way ANOVA followed by Tukey’s multiple comparison test. S3 Fig: Characteristics of naïve alveolar macrophages utilized for RNA sequencing studies. The (A) total cell count and (B) differential cell analysis was determined following bilateral whole lung lavage. The total number of cells present in the BAL was similar between wild type and HRB-Mertk-/- mice of both sexes. The majority of cells isolated in the BAL of wild type and HRB-Mertk-/- mice were airway macrophages with very few lymphocytes, polymorphonuclear leukocytes (PMN), or other cell types. Data were collected from two independent experiments and are expressed as mean ± SEM. (DOCX) [file pone.0320660.s001.docx]

**Supporting Information**

**Host responses to *S. pneumoniae* in wild type and Mertk mutant mice**

Matthew K. McPeek^1^, Jessica R. Martin^1^, John C. Gomez^1^, Yitong Li^2^, Hong Dang^1^, H. Shelton Earp^3^, Claire M. Doerschuk^1,4, 5^

**S1Table: Antibodies used in flow cytometry studies**

| **Flow Cytometry Antibodies – BAL Cell Panel** | | | |
| --- | --- | --- | --- |
| Antibody | Source | Clone | Concentration(µg/µL) |
| anti-CD3 eF450 | eBioscience | 48-003280 | 0.002 |
| anti-CD45 FITC | BD Biosciences | 553080 | 0.005 |
| anti-CD64 PE-Cy7 | BioLegend | 139314 | 0.002 |
| anti-Ly6C BV785 | BioLegend | 128041 | 0.002 |
| anti-Ly6G APC | BioLegend | 127614 | 0.002 |
| anti-Siglec F PE | BD Biosciences | 552126 | 0.002 |

| **Flow Cytometry Antibodies – Lung Homogenate Panel** | | | |
| --- | --- | --- | --- |
| Antibody | Source | Catalog Number | Concentration(µg/µL) |
| anti-CD45 FITC | BD Biosciences | 553080 | 0.005 |
| anti-CD64 PE-Cy7 | BioLegend | 139314 | 0.002 |
| anti-Ly6C BV785 | BioLegend | 128041 | 0.002 |
| anti-Ly6G APC | BioLegend | 127614 | 0.002 |
| anti-Mertk APC | BioLegend | 151507 | 0.002 |
| anti-Nk1.1 PE Dazzle | Biolegend | 108748 | 0.002 |
| anit-Siglec F PB | BD Biosciences | 565934 | 0.002 |

| **S2 Table: Original body weights, inoculum, and weight loss of animals infected with *S. pneumoniae.***  **A: 24-hour *S. pneumoniae* inoculation.** | | | | | |
| --- | --- | --- | --- | --- | --- |
| **Sex and Genotype** | ***n*** | **Original**  **Body Weight (g)** | **Number of**  ***S. pneumoniae*** | **Body weight**  **24h PI (g)** | **% Original**  **Body Weight** |
| Female wild type | 47 | 19.2 ± 0.1 | 0.85 ± 0.1 x10^6^ | 17.7 ± 0.1 | 92.0 ± 0.3 |
| Female HRB-*Mertk^-/-^* | 46 | 20.2 ± 0.2 | 0.91 ± 0.1 x10^6^ | 18.3 ± 0.2 | 91.0 ± 0.4 |
| Male wild type | 73 | 25.3 + 0.2 | 1.24 ± 0.5 x10^6^ | 23.1 ± 0.2 | 91.4 ± 0.3 |
| Male HRB-*Mertk^-/-^* | 70 | 25.2 ± 0.3 | 1.27 ± 0.5 x10^6^ | 22.9 ± 0.3 | 90.9 ± 0.3 |
|  |  |  |  |  |  |
|  | | | | | |
| **Select comparison** |  | **Original**  **Body Weight** | **Number of**  ***S. pneumoniae*** | **Body weight 24h PI** | **% Original Body Weight** |
| Female wild type **vs** female HRB-*Mertk^-/-^* |  | n.s. | n.s. | n.s. | n.s. |
| Male wild type **vs** male HRB-*Mertk^-/-^* |  | n.s. | n.s. | n.s. | n.s. |
| Female wild type **vs** male wild type |  | *p* < 0.01 | *p* < 0.01 | *p* < 0.01 | n.s. |
| Female *Mertk^-/-^* **vs** male *Mertk^-/-^* |  | *p* < 0.01 | *p* < 0.01 | *p* < 0.01 | n.s. |
|  |  |  |  |  |  |
| **B: IFN**γ **neutralization study.** | | | | | |
| **Genotype and Treatment** | ***n*** | **Original**  **Body Weight (g)** | **Number of**  ***S. pneumoniae*** | **Body weight 24h PI (g)** | **% Original Body Weight** |
| Wild type, PBS control | 9 | 18.6 ± 0.4 | 0.94 ± 0.2 x10^6^ | 16.9 ± 0.4 | 89.9 ± 0.3 |
| HRB-*Mertk^-/-^,* PBS control | 9 | 20.1 ± 0.5 | 1.00 ± 0.1 x10^6^ | 18.3 ± 0.4 | 91.5 ± 0.8 |
| Wild type, IgG control | 9 | 18.5 ± 0.4 | 1.08 ± 0.1 x10^6^ | 17.5 ± 0.3 | 94.7 ± 0.9 |
| HRB-*Mertk^-/-^,* IgG control | 9 | 19.2 ± 0.5 | 1.10 ± 0.1 x10^6^ | 17.6 ± 0.4 | 91.9 ± 0.6 |
| Wild type, anti-IFNγ | 9 | 17.3 ± 0.3 | 0.93 ± 0.1 x10^6^ | 16.3 ± 0.3 | 94.4 ± 0.9 |
| HRB-*Mertk^-/-^,* anti-IFNγ | 9 | 18.9 ± 0.3 | 1.01 ± **0.1** x10^6^ | 17.3 ± 0.2 | 91.4 ± 0.4 |
|  |  |  |  |  |  |
|  | | | | | |
| **Select comparison** |  | **Original**  **Body Weight** | **Number of**  ***S. pneumoniae*** | **Body weight 24h PI** | **% Original Body Weight** |
| PBS -wild type ***vs*** PBS-HRB*-Mertk^-/-^* |  | n.s. | n.s. | *p* < 0.05 | n.s. |
| IgG-wild type ***vs*** IgG-HRB-*Mertk^-/-^* |  | n.s. | n.s. | n.s. | n.s. |
| α-IFNγ-wild type ***vs*** α-IFNγ*-*HRB-*Mertk^-/-^* |  | *p=*0.04 | n.s. | n.s. | *p* = 0.04 |
| PBS -wild type ***vs*** IgG-wild type |  | n.s. | n.s. | n.s. | *p* < 0.01 |
| IgG-wild type ***vs*** α-IFNγ-wild type |  | n.s. | n.s. | n.s. | n.s. |
| PBS *-*HRB-*Mertk^-/-^* ***vs*** IgG*-*HRB-*Mertk^-/-^* |  | n.s. | n.s. | n.s. | n.s. |
| IgG*-*HRB-*Mertk^-/-^* ***vs*** α-IFNγ*-*HRB-*Mertk^-/-^* |  | n.s. | n.s. | n.s. | n.s. |

**S2 Table: Original body weights, inoculum, and weight loss of animals infected with *S. pneumoniae.*** The sample size (*n*), original body weights, number of *S. pneumonia* inoculated, body weight 24 hours post inoculation (24h PI) and percent original body weight of (**A**) animals evaluated in 24 hours post inoculation or (**B**) studies utilizing IFNγ neutralization, also studied 24 hours post inoculation (Fig 6). Statistically significant differences among each group were determined by one-way ANOVA followed by Bonferroni’s multiple comparisons test. The adjusted p values for selected comparisons and each metric are shown. Data represent mean ± SEM.

**S1 Fig: Representative gating strategy utilized for flow cytometric analysis of left lung homogenates**.


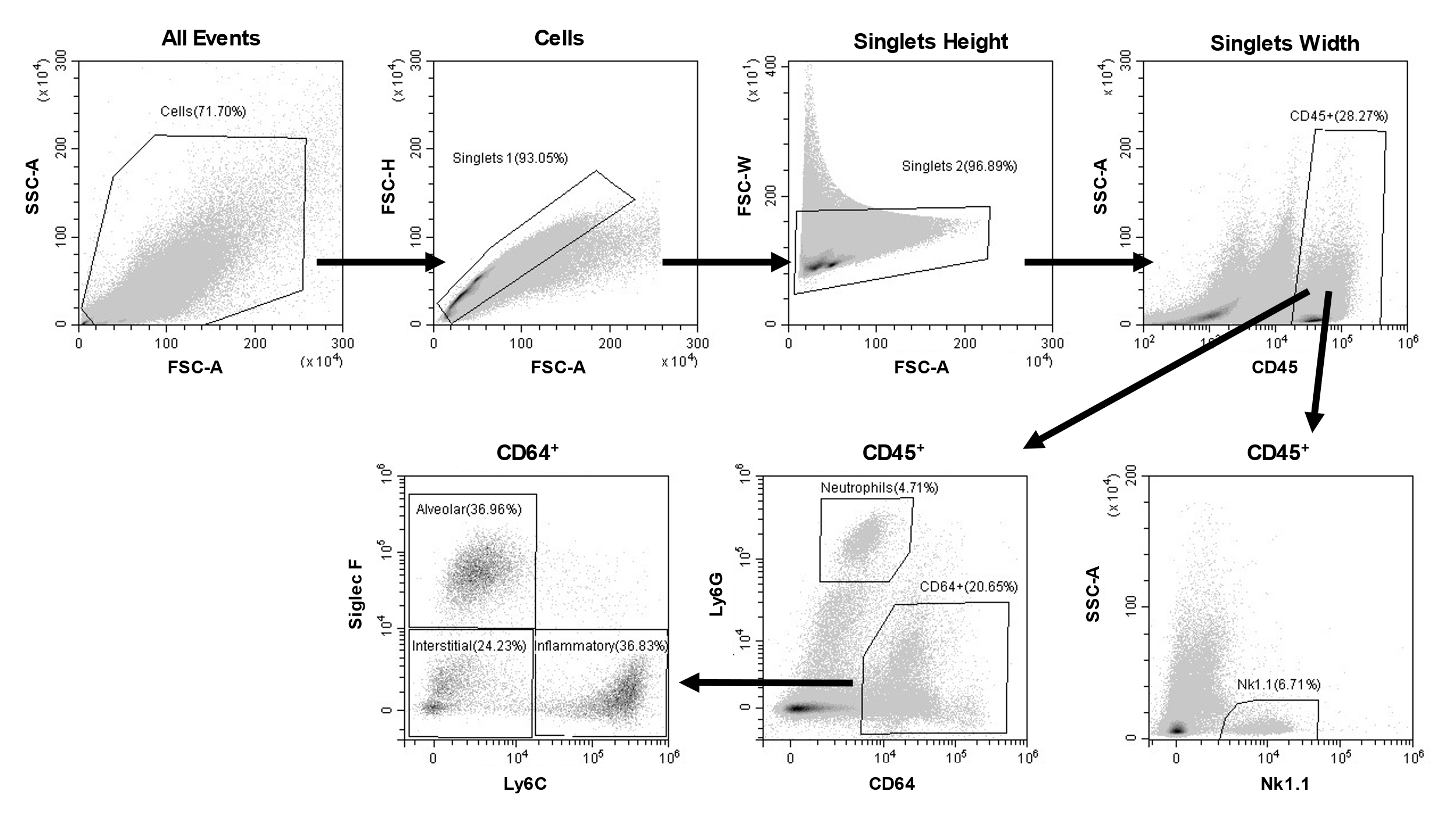


**S2 Fig: Characterization of immune cells present in the left lung of naïve HRB-*Mertk^-/-^* and wild type mice.**

**S2 Fig: Characterization of immune cells present in the left lung of naïve HRB-*Mertk^-/-^* and wild type mice.** The (**A**) number of natural killer cells present in left lung homogenates of naïve HRB-*Mertk^-/-^* and wild type mice were determined by flow cytometry. The (**B**) number of HRB-Mertk^+^ natural killer cells and (**C**) percentage of HRB-Mertk^+^ natural killer cells were determined. The (**D**) number of HRB-Mertk^+^ neutrophils and (**E**) percentage of HRB-Mertk^+^ neutrophils were derived from the total number of neutrophils (Fig 2C). Data are presented as mean ± SEM. Statistically significant differences evaluated by one-way ANOVA followed by Tukey’s multiple comparison test.

**S3 Fig: Characteristics of naïve alveolar macrophages utilized for RNA sequencing studies.**

******

**S3 Fig:** **Characteristics of naïve alveolar macrophages utilized for RNA sequencing studies.** The (**A**) total cell count and (**B**) differential cell analysis was determined following bilateral whole lung lavage. The total number of cells present in the BAL was similar between wild type and HRB-*Mertk^-/-^* mice of both sexes. The majority of cells isolated in the BAL of wild type and HRB-*Mertk^-/-^* mice were airway macrophages with very few lymphocytes, polymorphonuclear leukocytes (PMN), or other cell types. Data were collected from two independent experiments and are expressed as mean ± SEM.
